# Japanese clinical practice guidelines for vascular anomalies 2017

**DOI:** 10.1007/s11604-019-00885-5

**Published:** 2020-03-23

**Authors:** Hidefumi Mimura, Sadanori Akita, Akihiro Fujino, Masatoshi Jinnin, Mine Ozaki, Keigo Osuga, Hiroki Nakaoka, Eiichi Morii, Akira Kuramochi, Yoko Aoki, Yasunori Arai, Noriko Aramaki, Masanori Inoue, Yuki Iwashina, Tadashi Iwanaka, Shigeru Ueno, Akihiro Umezawa, Michio Ozeki, Junko Ochi, Yoshiaki Kinoshita, Masakazu Kurita, Shien Seike, Nobuyuki Takakura, Masataka Takahashi, Takao Tachibana, Kumiko Chuman, Shuji Nagata, Mitsunaga Narushima, Yasunari Niimi, Shunsuke Nosaka, Taiki Nozaki, Kazuki Hashimoto, Ayato Hayashi, Satoshi Hirakawa, Atsuko Fujikawa, Yumiko Hori, Kentaro Matsuoka, Hideki Mori, Yuki Yamamoto, Shunsuke Yuzuriha, Naoaki Rikihisa, Shoji Watanabe, Shinichi Watanabe, Tatsuo Kuroda, Shunsuke Sugawara, Kosuke Ishikawa, Satoru Sasaki

**Affiliations:** 1 Department of Radiology, St. Marianna University School of Medicine, 2-16-1, Sugao, Miyamae, Kawasaki, Kanagawa 216-8511 Japan; 2 Department of Plastic Surgery, Wound Repair and Regeneration, Fukuoka University, School of Medicine, Fukuoka, Japan; 3 Division of Surgery, National Center for Child Health and Development, Tokyo, Japan; 4 Department of Dermatology, Wakayama Medical University, Wakayama, Japan; 5 Department of Plastic and Reconstructive, Aesthetic Surgery, Kyorin University School of Medicine, Mitaka, Japan; 6 Department of Diagnostic and Interventional Radiology, Osaka University Graduate School of Medicine, Suita, Japan; 7 Department of Plastic Surgery, Ehime University Hospital, Toon, Japan; 8 Department of Pathology, Osaka University Graduate School of Medicine, Suita, Japan; 9 Department of Dermatology, Saitama Medical University, Irumagun, Japan; 10 Department of Medical Genetics, Tohoku University School of Medicine, Sendai, Japan; 11 Department of Plastic and Reconstructive Surgery, Keio University School of Medicine, Tokyo, Japan; 12 Department of Radiology, Keio University School of Medicine, Tokyo, Japan; 13 Department of Pediatric Surgery, The University of Tokyo Hospital, Tokyo, Japan; 14 Department of Pediatric Surgery, Tokai University School of Medicine, Isehara, Japan; 15 Department of Reproductive Biology, Center for Regenerative Medicine, National Center for Child Health and Development, Tokyo, Japan; 16 Department of Pediatrics, Gifu University Graduate School of Medicine, Gifu, Japan; 17 Department of Diagnostic Radiology, Tohoku University, Sendai, Japan; 18 Department of Pediatric Surgery, Niigata University Graduate School of Medical and Dental Sciences, Niigata, Japan; 19 Department of Plastic and Reconstructive Surgery, The University of Tokyo Hospital, Tokyo, Japan; 20 Department of Plastic Surgery, Osaka University Graduate School of Medicine, Suita, Japan; 21 Department of Signal Transduction, Research Institute for Microbial Diseases, Osaka University, Suita, Japan; 22 Department of Dermatology, Osaka Red Cross Hospital, Osaka, Japan; 23 Department of Dermatology, Kanto Central Hospital, Tokyo, Japan; 24 Department of Radiology, Kurume University School of Medicine, Kurume, Japan; 25 Department of Plastic and Reconstructive Surgery, Graduate School of Medicine, Mie University, Tsu, Japan; 26 Department of Neuroendovascular Therapy, St. Luke’s International Hospital, Tokyo, Japan; 27 Division of Radiology, National Center for Child Health and Development, Tokyo, Japan; 28 Department of Radiology, St Luke’s International Hospital, Tokyo, Japan; 29 Department of Plastic and Reconstructive Surgery, Juntendo University Urayasu Hospital, Urayasu, Japan; 30 Department of Dermatology, Hamamatsu University School of Medicine, Hamamatsu, Japan; 31 Department of Pathology, Dokkyo Medical University, Saitama Medical Center, Koshigaya, Japan; 32 Department of Plastic and Reconstructive Surgery, Shinshu University School of Medicine, Matsumoto, Japan; 33 Department of Plastic and Reconstructive Surgery, Oyumino Central Hospital, Chiba, Japan; 34 Department of Plastic and Reconstructive Surgery, Saitama Children’s Medical Center, Saitama, Japan; 35 Department of Dermatology, Teikyo University School of Medicine, Tokyo, Japan; 36 Department of Pediatric Surgery, Keio University School of Medicine, Tokyo, Japan; 37 Department of Diagnostic Radiology, National Cancer Center Hospital, Tokyo, Japan; 38 Department of Plastic and Reconstructive Surgery, Faculty of Medicine and Graduate School of Medicine, Hokkaido University, Sapporo, Japan; 39 Department of Plastic and Reconstructive Surgery, Center for Vascular Anomalies, Tonan Hospital, Sapporo, Japan

**Keywords:** Clinical practice, Guidelines, Hemangioma, Vascular anomalies, Vascular malformation

## Abstract

The objective was to prepare guidelines to perform the current optimum treatment by organizing effective and efficient treatments of hemangiomas and vascular malformations, confirming the safety, and systematizing treatment, employing evidence-based medicine (EBM) techniques and aimed at improvement of the outcomes. Clinical questions (CQs) were decided based on the important clinical issues. For document retrieval, key words for literature searches were set for each CQ and literature published from 1980 to the end of September 2014 was searched in Pubmed, Cochrane Library, and Japana Centra Revuo Medicina (JCRM). The strengths of evidence and recommendations acquired by systematic reviews were determined following the Medical Information Network Distribution System (MINDS) technique. A total of 33 CQs were used to compile recommendations and the subjects included efficacy of resection, sclerotherapy/embolization, drug therapy, laser therapy, radiotherapy, and other conservative treatment, differences in appropriate treatment due to the location of lesions and among symptoms, appropriate timing of treatment and tests, and pathological diagnosis deciding the diagnosis. Thus, the Japanese Clinical Practice Guidelines for Vascular Anomalies 2017 have been prepared as the evidence-based guidelines for the management of vascular anomalies.

## Introduction

The etiology of vascular anomalies on the body surface and in soft tissue are mostly unclear and no fundamental treatment methods have been established. Many patients visit many medical institutions seeking an expert, being a disadvantage in treatment. Hemangiomas and vascular malformations are frequently termed ‘hemangioma’, but these are different diseases in the ISSVA classification proposed by the International Society for Study of Vascular Anomalies (ISSVA) [1, 2], and this classification has been internationally standardized.

‘Clinical Practice Guidelines for Vascular Anomalies 2013’ (1st edition) [3] target general practitioners and the general public, and were prepared aiming at organizing effective and efficient treatments for hemangiomas/vascular malformations, confirming the safety, and systematizing treatment using evidence-based medicine (EBM) techniques. The organization responsible for preparation was the Health, Labour and Welfare Sciences Research Grants (Research on Measures for Intractable Diseases), Research Committee for ‘Intractable Vascular Anomalies’, and the main committee members were selected from academic societies of plastic surgery and radiology mainly treating hemangiomas and vascular malformations: the Japanese Society of Plastic and Reconstructive Surgery and Japanese Society of Interventional Radiology, and the guidelines were prepared by them.

‘Clinical Practice Guidelines for Vascular Anomalies 2017’ were prepared as a revised edition of the ‘Clinical Practice Guidelines for Vascular Anomalies 2013’. The organization responsible for preparation was the Health, Labour and Welfare Sciences Research Grants (Research on Policy Planning and Evaluation for Rare and Intractable Diseases), Research Committee for Intractable Vascular Anomalies, and the differences from the previous guidelines are setting the objective at summarizing opinions from related academic societies by inviting many committee members from dermatologists, pediatric surgeons, pediatricians, radiologists (diagnostic radiology), and basic researchers including the pathology, molecular biology, and epidemiology fields, in addition to plastic surgeons and radiologists (interventional radiology). Since the guidelines were prepared following the reformed ‘Minds Handbook for Clinical Practice Guideline Development 2014’ [4] and ‘Minds Manual for Clinical Practice Guideline Development Ver.1.0-2.0’ [5, 6], it was fully revised.

The original text of the guidelines (Japanese version) is comprised of Reviews and Clinical Questions (CQs), but only CQs are presented in this report.

## Purpose of the guidelines

The objective was to prepare guidelines to perform the current optimum treatment by organizing effective and efficient treatments of hemangiomas and vascular malformations, confirming the safety, and systematizing treatment, employing the EBM techniques and aimed at improvement of the following outcomes: Pain, swelling, esthetic impairment, and functional disorder.

## Materials and methods

### Organization

For the Guidelines Executive Committee members, representatives of the plastic surgery, dermatology, radiology, pediatric surgery, and basic science fields were selected. The guidelines preparation group and systematic review team for preparation of CQs and recommendations were comprised of 4 groups: groups in charge of arteriovenous malformation (AVM), venous malformation (VM) , combined type, and syndrome, in charge of capillary malformation (CM) and infantile hemangioma, in charge of the lymphatic malformation (lymphangioma) (LM), and in charge of the basic field. To the group in charge of AVM, VM, combined type, and syndrome, plastic surgeons and radiologists were mainly assigned. To the group in charge of CM and infantile hemangioma, plastic surgeons and dermatologists were mainly assigned. To the group in charge of the lymphatic system, pediatric surgeons, plastic surgeons, and pediatricians were mainly assigned. The Reviews of the guidelines were also prepared by those selected from each group. Pathologists and molecular biologists were in charge of the Reviews of the basic fields.

### Preparation process

The guidelines were revised following the ‘Minds Handbook for Clinical Practice Guideline Development 2014’ and ‘Minds Manual for Clinical Practice Guideline Development Ver.1.0-2.0’.

CQs were decided based on the following important clinical issues: (1) Efficacy of resection, (2) efficacy of sclerotherapy/embolization, (3) efficacy of drug therapy, laser therapy, radiotherapy, and other conservative treatment, (4) differences in appropriate treatment due to the location of lesions, (5) differences in appropriate treatment among symptoms, (6) appropriate timing of treatment and tests, and (7) pathological diagnosis deciding the diagnosis.

For document retrieval, key words for literature searches were set for each CQ and literature published from 1980 to the end of September 2014 was searched in Pubmed, Cochrane Library, and Japana Centra Revuo Medicina (JCRM). Literature search was requested to the Japan Medical Library Association. For decisions on CQs and recommendations lacking evidence or having weak evidence, discussion and agreement in the preparation group were reflected.

The strengths of evidence and recommendations acquired by systematic reviews were determined following the Minds technique as described below and this follows the GRADE guidelines preparation method [7, 8].

### Determination of the strength of evidence of the body of evidence (Table 1)

The strength of evidence of the body of evidence was determined according to ‘Minds Handbook for Clinical Practice Guideline Development 2014’.

**Table 1:** Recommendation grade and definition of the strength of body of evidence in evaluation of systematic review

Recommendation grade
1 strongly recommended
2 weakly recommended (suggested)
Definition of the strength of body of evidence in evaluation of systematic review
A (strong): strongly confident of the estimate of effect
B (moderate): moderately confident of the estimate of effect
C (weak): limited confidence of the estimate of effect
D (very weak): very little confident of the estimate of effect

In the case of RCTs, the score “A (strong)” is given at the start of evaluation, and the final score might be downgraded to B, C, or D, according to the results of evaluation of five items, including risk of bias, inconsistency in results, indirectness of evidence, data imprecision, and high possibility of publication bias. In the case of observational studies, the score “C (weak)” is given at the start of evaluation, and five items lowering the strength are evaluated similarly as for RCTs. In addition, three items, including large effect with no confounding factors, dose–response gradient, and possible confounding factors, are weaker than actual effects increasing the strength are evaluated as well.

### Presentation of the strength of recommendations (Table 1)

The strength of recommendation was also determined according to ‘Minds Handbook for Clinical Practice Guideline Development 2014’.

The strength of recommendations is usually presented in two ways: “1”: strongly recommended, and “2”: weakly recommended (suggested). If the strength of recommendations cannot be determined by any means, it is occasionally presented as “no definite recommendation can be made”. Recommendations will be entered as follows by indicating the strength of evidence (A, B, C, D) with the strength of recommendations “1”: strong or “2”: weak.

### Finalization

Preparation of the draft guidelines was completed in December 2016 and review was requested to the Japanese Society of Plastic and Reconstructive Surgery, Japanese Dermatological Association, Japan Radiology Society, Japanese Society of Interventional Radiology, Japanese Society of Pediatric Surgeons, and Japanese Society of Pathology between December 2016 and January 2017, and corrections were made based on the results of the reviews. In addition, from December 2016 to January 2017, the guidelines were disclosed on the home page of the Research Committee for ‘Intractable Vascular Anomalies’ and public comments were collected. The draft guidelines were presented to two related patient organizations, ‘the Patients Association of Vascular Anomalies’ and ‘the Patients Association of Combined Vascular Malformations’ and comments were received. Based on these, the draft guidelines were revised and CQs, recommendations, and explanations were completed. It was finalized in March 2017.

## Results

### CQs and recommendations


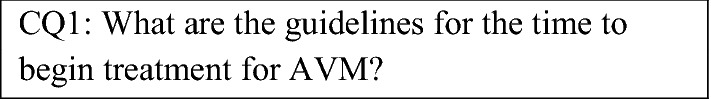


Recommendation:

It is necessary to judge the time to begin endovascular or surgical treatment for AVM individually by evaluating the stage of symptoms and lesion extent and in consideration of the risk of complications.

Strength of recommendation: 2 (weak)
Evidence: D (very weak)

#### Comments

As a result of primary screening, 92, 3, and 27 papers were extracted from PubMed, JCRM, and Cochrane Library, respectively, and, as a result of secondary screening, 37 and 3 papers were extracted from PubMed and JCRM, respectively. However, as all these references were observational or case series studies, the strength of evidence is rated as “D (very weak)”.

There has been no report in which the time to begin treatment for AVM in itself was the endpoint, and only some reports described the view on the time to begin treatment in the discussion. Therefore, as it is difficult to objectively evaluate the validity of the time to begin treatment, we determined whether guidelines can be derived from the patient age, lesion site, symptoms, clinical stage, effectiveness of treatment, frequency of complications in each report.

In the reports [9, 10] on the treatment for AVM, symptomatic AVM is primarily addressed, and treatment can be suspended (follow-up) while the lesion remains asymptomatic. However, as AVM often progresses when left untreated, it is considered important to begin the treatment at an appropriate time depending on the stage of symptoms. In addition, as there is the tendency that the response rate decreases, and the complication rate increases, with progression of symptoms, some reports from particular pediatric institutions where patients are concentrated recommend early therapeutic intervention in relatively “early” or “mild” stages without waiting for progression of the disease.

Localized lesions may be radically treated by early intervention [11]. Among endovascular procedures, the response (cure) rate tends to be high by ethanol embolization, but as the complication rate is also high [12, 13]. By surgery, localized lesions are unlikely to recur if they are completely resected, although adverse events, such as postoperative cicatrization/deformation or functional impairment, have been seldom discussed [10]. On the other hand, in diffuse lesions, limitations of effectiveness, such as recurrence and persistence and the risk of treatment, are higher by both endovascular treatment and surgery, with the risks outweighing benefits [13]. It should also be considered that children, in particular, are not mentally ready to accept such invasive treatments [14].

As discussed above, it is difficult to give guidelines for the time to begin treatment for AVM at present, and individual judgment is necessary depending on the symptom stage and lesion extent in consideration of the complication risk.


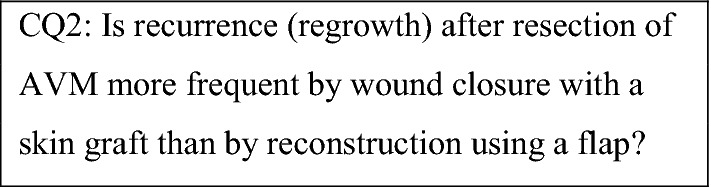


Recommendation:

Whether recurrence (regrowth) is more frequent by wound closure with a skin graft compared with reconstruction using a flap is unclear.

Strength of recommendation: 2 (weak)
Evidence: D (very weak)

#### Comments

The number of references retrieved by searches using keywords was 40 from JCRM, 75 from PubMed, and 0 from Cochrane, and 39 were extracted by secondary screening. For AVM of a certain size, reconstruction is necessary after resection, and wound closure by skin grafting or reconstruction using a flap is performed according to common reconstruction methods for tissue defects. The reports discussing reconstruction after resection of AVM that we could retrieve were all descriptive studies (case reports or case series studies). Therefore, the evidence level of all these references is D (very weak).

The concept of regulating flap [15, 16], that reconstruction using a free flap controls the recurrence or regrowth after resection of AVM, has been proposed. However, no report has evaluated whether free flaps [16–37] and other flap types [17, 19, 25, 28, 38–49] clearly prevent the recurrence or regrowth compared with skin grafts [21, 23, 50–52].

According to the present knowledge about the recurrence or regrowth after resection of AVM [15, 16, 39, 53], whether AVM can be completely resected is important, and, concerning cases in which complete resection is difficult, it has been reported that the hemodynamics in the residual lesions contributes to the recurrence and regrowth, and that it can be controlled by a flap with rich blood flow.


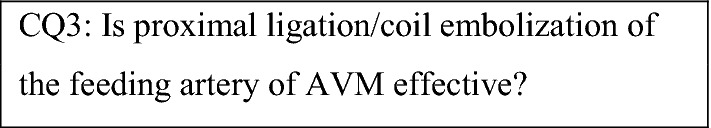


Recommendation:

The therapeutic effect of ligation/coil embolization of the feeding artery on the proximal side may be poor, and the possibility of recurrence may be high. In addition, in the event of recurrence, treatment may become difficult due to the development of collateral vessels. Therefore, these procedures are recommended to be avoided, in principle.

Strength of recommendation: 2 (weak)
Evidence: D (very weak)

#### Comments

As a result of secondary screening, 1 and 14 papers were extracted from PubMed and JCRM, respectively, and were reviewed. As a result, all these papers were case reports. In addition, 6 papers retrieved by manual searches were also reviewed, but they were all case series studies at the maximum. Therefore, the strength of evidence as a collection of literature concerning this CQ is D (very weak).

To summarize the evaluation of this collection of papers, AVM was treated by proximal ligation/coil embolization of the feeding artery, but as there have been reports of recurrence following the development of collateral channels (reports of cases of the occurrence of unfavorable situations), the treatment is recommended to be avoided, but the evidence level of this recommendation is low as mentioned above.

The objective of embolization for AVM is obliteration of the nidus, and embolization at or near the nidus is necessary as much as possible. If ligation/coil embolization of the feeding artery is performed on the proximal/central side, the nidus is not obliterated, and the development of several collateral channels is promoted. In many cases, the collateral channels are thin, complicated, and markedly tortuous, and trans-catheter treatment is often difficult.

Wu et al. [14] performed proximal ligation in 9 of 29 patients treated for AVM of the auricle but that the condition was exacerbated in all patients with 8 requiring auricular resection and 1 requiring additional treatment. They excluded proximal ligation from treatment options for AVM, because it makes subsequent trans-catheter treatments difficult.

Slaba et al. [54] evaluated 25 patients with AVM of the tongue and reported that 3 of the 12 symptomatic patients who underwent ipsilateral external carotid artery ligation at another facility showed marked development of collateral channels.

Other reports include those of a case in which a large number of collateral channels developed as a result of ligation of the feeding artery for AVM of the shoulder with serious complications including high-output heart failure [55], 3 cases in which proximal ligation/embolization was performed for AVM of the limbs and pelvis, collateral vessels developed, but the condition was controlled by multidisciplinary treatment consisting of trans-catheter treatment and direct puncture sclerotherapy [56], and several cases in which external carotid artery ligation was performed for AVM of the head and neck, but the subsequent treatment was difficult.

Suyama et al. [35] reported a case in which AVM of the auricle, treated by proximal embolization using coils and gelatin sponge, recurred and was treated again by ligation at a proximal part of the artery, but the lesion recurred again. Also, Aikawa et al. [57] reported a case of intrapelvic AVM that underwent coil embolization of the left ovarian artery and left internal iliac artery but showed little change in the area of the nidus or the state of arterial or venous dilatation. In addition, Yamamoto et al. [58] reported a case in which TAE was performed for AVM of the mandible via the maxillary artery, facial artery, lingual artery, and ocular artery but was not effective due to the development of collateral channels from the internal carotid artery and vertebral artery.

As observed above, it is recommended not to select proximal/central ligation/coil embolization as a treatment for AVM. However, AV fistulas with direct connection of a large artery and a vessel may be treated by coil embolization if the shunt area is directly accessible with a catheter. Proximal coil embolization may also be accepted as preoperative embolization, but careful evaluation of its indications is necessary, and embolization at a site near the shunt is necessary to leave the room for catheter insertion in the event of future recurrence.


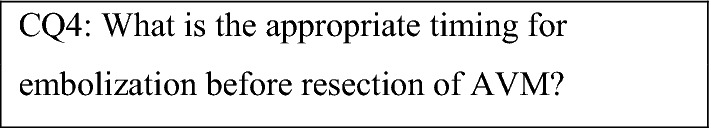


Recommendation:

It is recommended to perform resection within 3 days (72 h) after embolization. If the interval prolongs, the risk of massive intraoperative hemorrhage may increase due to recanalization of the embolized vessel and development of collateral channels. In addition, surgery has been reported to be made difficult by enlargement of the lesion after embolization.

Strength of recommendation: 2 (weak)
Evidence: D (very weak)

#### Comments

While it is difficult to generalize the therapeutic approach as it varies with the affected area and extent of the lesion, there were a few reports that preoperative embolization was useful for the treatment of AVM of the head and neck region.

As a result of secondary screening, 10 and 3 papers from PubMed and JCRM, respectively, were reviewed. All the papers selected by this screening procedure were case reports or case series, and the strength of evidence is “D (very weak)”. Mentions about the timing of preoperative embolization and volume of hemorrhage also varied among the papers. Although it is difficult to draw a conclusion, among the papers that mentioned specific timing of preoperative embolization and volume of hemorrhage, Deng et al. [59] performed embolization within 48–72 h before surgery in 16 patients with maxillofacial AVM and reported that the volume of hemorrhage was ≤ 200 mL in all patients and that there were no complications. Erdmann et al. [60] performed embolization within 24 h before surgery in 4 patients with head and neck AVM, and the lesion was resected with hemorrhage of ≤ 100 mL in 3. It is recommended to perform resection within 72 h to prevent increases in difficulty of resection due to inflammation after embolization.

There have also been reports that embolization was performed intraoperatively or within a few days before surgery, resulting in decreases in the volume of hemorrhage or favorable long-term outcomes. Most papers reported no or only mild complications, but as for relatively severe complications, Goldberg et al. [61] reported temporary visual impairment in 2 of the 3 patients with orbital AVM.

Factors that affect the appropriate timing of preoperative embolization include recanalization of the target vessel, development of collateral channels, and swelling and reactive changes after embolization, which make surgery difficult. To avoid the effects of these phenomena, many papers supported relatively early resection, i.e., within 72 h after embolization. Clinically, also, there is no benefit in taking a long interval, and it is considered valid to recommend resection within 72 h after embolization.

In conclusion, adequate control of hemorrhage may be achieved with fewer complications by performing vascular embolization within a few days before surgery, but no sufficient evidence that support this view has been provided.


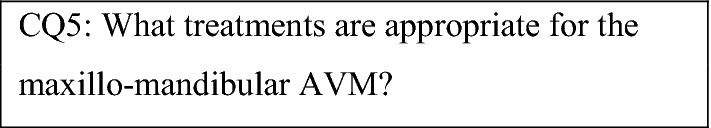


Recommendation:

Although surgery alone is not recommended, a combination of surgery with endovascular embolization (including sclerotherapy) can be recommended depending on the case.

Radiotherapy is not recommended.

Endovascular embolization (including sclerotherapy) alone or as a preoperative treatment can be recommended.

Strength of recommendation: 2 (weak)
Evidence: D (very weak)

#### Comments

AVM of the maxilla and mandible is a rare disorder. Most of the literature is reports of a small number of cases except a few case series reported from some special institutions. Only 5 reports [62–66] of a series of 10 or more cases were retrieved by the search of PubMed. Because there is no cohort study or randomized trial comparing various treatments, strong evidence regarding the best treatment is lacking.

Maxillo-mandibular AVM may involve the maxilla, mandible, or both, and it often presents with massive oral hemorrhage around the age of 10 years when milk teeth are lost, but may also be detected due to swelling of the soft tissue.

According to Persky et al. [62], embolization alone resulted in cure in 42%, improvement in 16%, and stabilization of symptoms in 23% of the 26 patients with a maxillo-mandibular AVM. Liu et al. [63] treated 25 patients by transarterial or transvenous embolization alone or in combination with curettage and reported anatomical cure in 14 and clinical cure in 21. Chen et al. [65] treated 15 patients by bone wax packing (BWP) alone in 4, transarterial embolization (TAE) + BWP in 3, TAE + resection in 4, and TAE + radiotherapy + resection in 4 and reported clinical cure in 14.

The following are considered as treatment options for AVM of the maxilla and mandible.

A. Surgical treatment
  1. Resection and reconstruction
  2. Curettage
  3. Bone wax packing
B. Endovascular embolization (including sclerotherapy)
  1. Transarterial embolization
  2. Transvenous embolization
  3. Embolization by direct puncture
  4. Combination of A and B
  5. Radiotherapy

The literature is mostly about B, i.e., endovascular embolization (including sclerotherapy) alone, or surgical treatment after B. There was no report of case series of surgical treatment alone, but there was only 1 report [65] of case series of surgery + radiotherapy. Surgery alone and radiotherapy are generally not recommended. Endovascular embolization is performed by various approaches including transarterial and transvenous routes and direct puncture, sometimes, in combination. Concerning embolic agents, PVA and Gelfoam^®^ (Pfizer, New York, NY, USA) are used for embolization as an adjuvant therapy immediately before surgery as they allow recanalization. Cyanoacrylate liquid embolic agents are considered effective for embolization performed preoperatively or alone in expectation of a long-term occlusive effect [64, 67, 68]. Coils are often used for transvenous embolization. Recently, there have been reports [69, 70] that favorable outcomes were obtained by TAE using Onyx^®^ (Medtronic, Irvine, CA, USA), a non-adhesive liquid embolic agent. Concerning sclerotherapy, there is a case series study of ethanol sclerotherapy alone, reporting relatively favorable outcomes [71]. Infection and bone necrosis are frequent complications of embolization, and they may occur, when an embolic agent, a foreign body, is injected into lesions that were in contact with the external environment due to direct puncture or hemorrhage. Surgical treatments as listed above are performed primarily after endovascular embolization. Invasive radical resection and reconstruction should be avoided at least as the initial treatment, because many lesions can now be controlled by endovascular embolization alone with the advancement of endovascular technique.

As mentioned above, endovascular embolization is performed using various approaches and embolic agents selected depending on the facility and patient. There are also a wide variety of surgical treatment options. Since the treatment may be performed by combining these options, AVM of the maxilla and mandible should be treated at the institutions where multidisciplinary treatment can be performed by experienced physicians.


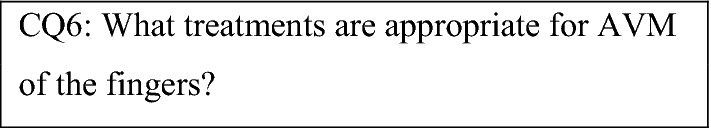


Recommendation:

Although embolization or sclerotherapy is effective as it alleviates symptoms, such as pain, sufficient evaluation is necessary because of the risk of finger necrosis and nerve damage. In surgical resection, total resection is recommended, because partial resection is likely to result in enlargement of the lesion. Occasionally, the disorder results in finger amputation.

Strength of recommendation: 2 (weak)
Evidence: D (very weak)

#### Comments

As a result of primary screening, 38, 16, and 35 papers were retrieved from PubMed, Cochrane Library, and JCRM, respectively. However, during secondary screening, many reports concerned AV shunts in dialysis patients and AVM at sites other than the fingers. Eventually, only 10 papers consisting of 3 case series and 7 case reports remained as references, and the evidence level is extremely low (D: very weak).

AVM of the fingers is often difficult to treat, and treatments are likely to be ineffective, particularly, when the lesion extends from the fingers to the palm. In addition, when AVM is localized in the fingers, complications are likely to occur after treatment [72]. It is recommended to conduct treatment by a team from several departments including the plastic surgery, vascular surgery, and radiology departments [73]. 3D-CTA is useful for preoperative examination [74]. Since complete cure is difficult to obtain by embolization therapy, it is recommended to be performed for alleviation of symptoms such as pain only in symptomatic areas [75]. In addition, as there is the possibility of re-enlargement after embolization, it is recommended to periodically follow-up the condition and repeatedly perform embolization each time symptoms appear [73]. Surgical resection is necessary for permanent cure, and total resection is recommended as there is the possibility of re-enlargement after partial resection [76–78]. Reconstruction is occasionally necessary, but treatment may end in finger amputation. In this event, preoperative embolization or sclerotherapy is effective [79]. The present review has fallen short of clarifying situations in which preoperative embolization is useful in fingers to which a tourniquet can be applied.


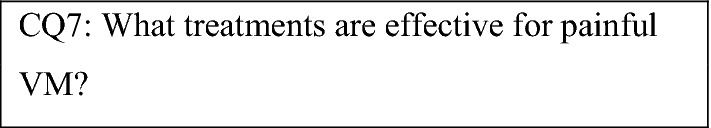


Recommendation:

Sclerotherapy and surgical resection, as well as conservative treatments, such as compression, oral aspirin, and low-molecular-weight heparin, are reported to be effective depending on the site and size of the lesion and symptoms. Endovascular laser treatment, percutaneous cryotherapy, and photodynamic therapy have also been suggested to be effective.

Strength of recommendation: 2 (weak)
Evidence: D (very weak)

#### Comments

As a result of literature search, 54 reports in English and 4 in Japanese were retrieved by primary screening. Of these reports, 39 in English and 4 in Japanese were extracted by secondary screening. Many options were reported as treatments for pain associated with VM, but all these documents were case series or case reports without comparison of treatments. Therefore, the evidence level was rated as “very weak”, and the recommendation level as “weak”.

Pain is one of the major symptoms of VM. It may respond to conservative treatments that are relatively easy to manage, such as compression and oral aspirin, depending on the site and size of the lesion and symptoms. Particularly, when pain is localized, surgery should also be considered. Relatively novel treatments, such as endovascular laser therapy, percutaneous cryotherapy, and photodynamic therapy, have been reported to be effective for controlling local VM, and they have also been reported to be effective for the control of pain. Limb lesions accompanied by localized intravascular coagulopathy may be indications for low-molecular-weight heparin. Reports on various treatments are mentioned below.

1. Compression

Although there has been no report of comparative evaluation, compression is reportedly effective according to reviews by specialized medical facilities [80–82].

2. Oral aspirin

The literature is also limited, but the treatment has been mentioned in reviews [80–82]. Nguyen et al. [83] reported that pain was alleviated in 17 (77%) of 22 patients in whom oral aspirin therapy was initiated for pain.

3. Sclerotherapy

Sclerotherapy has often been performed using ethanol or polidocanol. The literature concerning other sclerosing agents is scarce, and their effectiveness remains largely unclear.

Each sclerosing agent is commented below.

i. Ethanol

Shireman et al. [84] reported remission in 6 of 12 patients, and Rimon et al. [85] reported alleviation or remission in 14 patients with painful VM (including 8 with lower limb lesions) except in 4 of those with lower limb lesions. Marrocco-Trischitta et al. [86] reported that pain was resolved in both (100%) of 2 women with external genital lesions.

Concerning the use of ethanol, Suh et al. [87] reported alleviation to 50% or less of the pre-treatment state according to a VAS in 12 (71%) of 17 patients who underwent sclerotherapy using its mixture with lipiodol, and Dompmartin et al. [88] reported 37 patients who underwent sclerotherapy using its mixture with ethylcellulose. According to Schumacher et al. [89], also, 77 patients underwent sclerotherapy using ethylcellulose-ethanol in a multicenter study, and significant improvement compared with the pre-treatment state was observed in all patients.

ii. Polidocanol (including foam sclerotherapy)

Mimura et al. [90] reported remission in 6, alleviation in 4, and no change in 1 of 11 patients with painful VM, and remission in 12 (41%), alleviation in 14 (48%), no change in 2 (7%), and exacerbation in 1 (3%) of 29 patients in another study [91]. Cabrera et al. [92] treated 50 patients (including 15 with Klippel–Trenaunay syndrome) using a foamed sclerosing agent and reported remission in 25 (50%) and alleviation in 14 (28%). Marrocco-Trischitta et al. [86] reported resolution of pain in all 3 women with external genital lesions.

iii. Ethanolamine oleate

Ozaki et al. [93] reported remission in 2 (20%) and alleviation in 8 (80%) of 10 patients.

iv. Sodium tetradecyl sulfate

Krokidis et al. [94] reported alleviation of pain in 4 of 5 women with external genital lesions.

4. Surgical resection

Enjolras et al. [95] performed surgical resection in 7 of 13 patients with VM involving a wide area including the knee joint and reported alleviation of pain in 5 (71%). Steiner et al. [96] reported alleviation to 50% or less of the pre-treatment state by a VAS in 24 (89%) of 27 patients with background pain and 12 (92%) of 13 patients with acute episodic pain. In addition, Noel et al. [97] performed surgical resection and compression therapy for VM of the lower extremities in 20 patients with Klippel–Trenaunay syndrome and reported disappearance of pain in 18 (90%) (mean follow-up period: 63 months).

5. Endovascular laser therapy

Sidhu et al. [98] and Lu et al. [99] reported alleviation of pain in all 8 and 51 lesions in 6 and 33 patients, respectively. Liu et al. [100] also reported marked responses in 46 (35%), responses in 84 (63%), and no change in 3 (2%) of 133 patients.

6. Low-molecular-weight heparin

According to Mazoyer et al. [101], only low-molecular-weight heparin was effective when VM are complicated by localized intravascular coagulation, resulting in disappearance of pain.

7. Percutaneous cryotherapy

Cornelis et al. [102, 103] reported remission of pain in a report of 1 case (observation period: 2 months) and a report of 4 cases (observation period: 6 months).

8. Photodynamic therapy

Betz et al. [104] reported remission in 2 and alleviation in 1 of 3 patients.


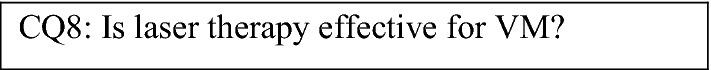


Recommendation:

With appropriate selection of the type of laser according to the site, size, and symptoms of the lesion, laser therapy can be effective for the treatment of VM. It is recommended to evaluate whether the net benefit by laser therapy is worth the cost and resources by comparison with other treatments such as sclerotherapy and surgical resection.

Strength of recommendation: 2 (weak)
Evidence: C (weak)

#### Comments

Venous malformation is a lesion that has been called cavernous hemangioma, and it causes pain, functional impairment, and cosmetic defect depending on the affected site. In addition to conventional resection of the lesion, sclerotherapy has been widely performed in recent years. While reports of laser therapy for VM have increased, there have not been prospective studies comparing the results of laser therapy with those of surgery or sclerotherapy, among types of laser equipment different in wavelength, or using the same equipment type but changing the irradiation method or parameter setting. We analyzed 134 papers extracted by primary screening and 98 papers extracted by secondary screening. Concerning more than 30 cases, the answer to the CQ was based primarily on 7 reports summarizing the methods and sites of treatment and benefits and harms derived from treatment (decrease in size of the lesion, alleviation of symptoms, complications).

In the facial skin, pigmentation and scar formation after irradiation can be serious treatment-related complications compared with unexposed areas. In the airway and digestive tract, the mass effect of the lesion and chronic bleeding from the lesion can be causes of serious symptoms. Thus, as the goal to achieve varies with the anatomical site of the lesion, we reviewed the literature by the anatomical site (the neurosurgery field was excluded). For this reason, we also extracted benefits and harms of treatment from the text of the reports of less than 30 cases. The relevant departments surveyed included ENT, dental and oral surgery, gastrointestinal surgery, ophthalmology, plastic surgery, and dermatology, and secondary screening overviewed laser therapy for VM and vasodilatory lesions.

**Fig. 1:**
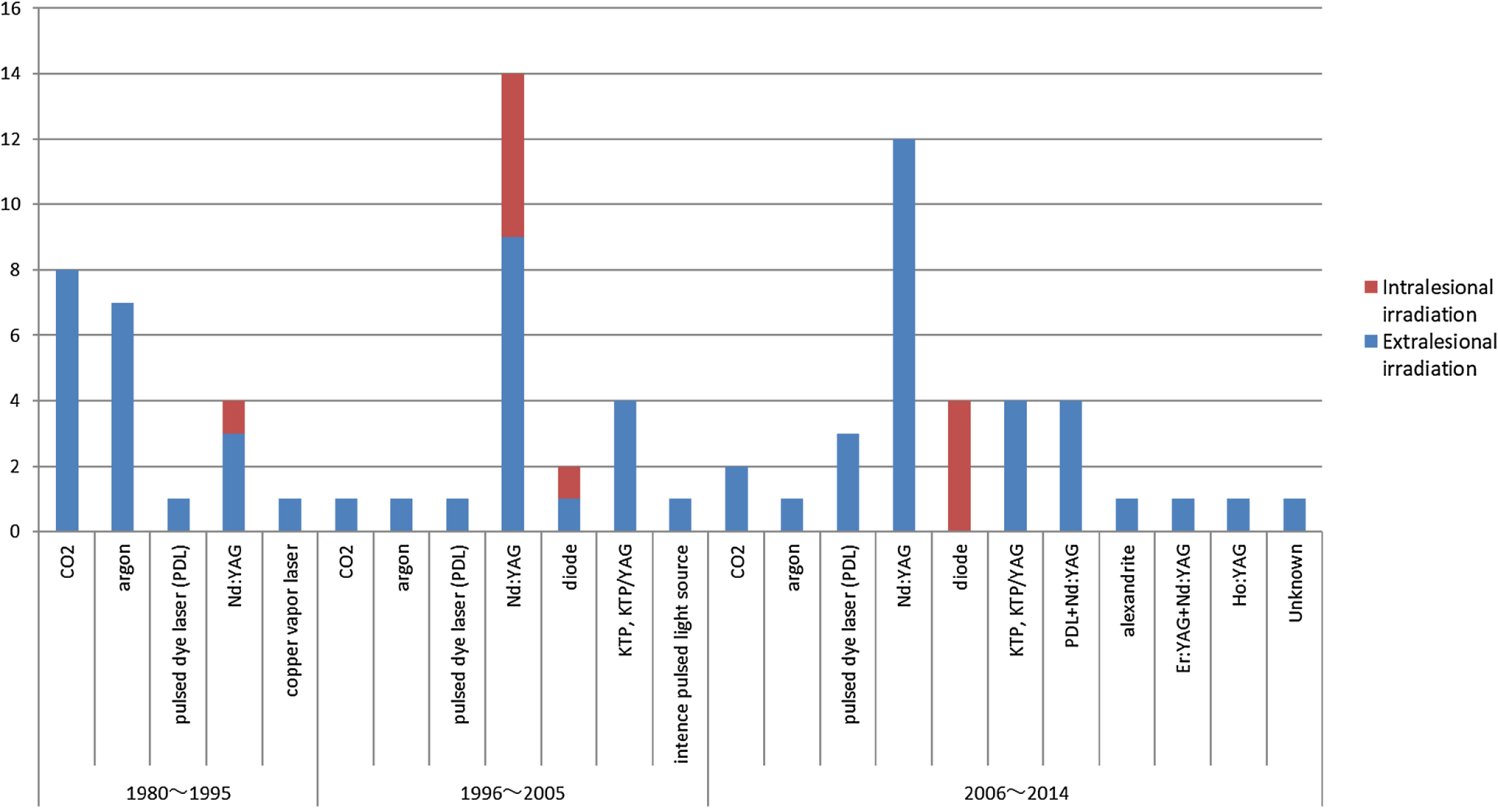
Reports of laser therapy for hemangiomas/vascular malformations (primarily venous) in various periods and types of laser used in the reports. CO_2_; 10 of 11 reports recommend the use for surgical resection. Argon; There are 4 reports of mixed treatments for CM and VM. Nd:YAG; There are 12 reports of its use in combination therapy with surgery, sclerotherapy, or other lasers. Alexandrite; There is only 1 report summarizing cases treated with alexandrite in combination therapy with other lasers

When a new laser instrument is developed and put into use, reports of therapeutic results using the equipment are presented. The types of laser used for treatment varied widely. The types of laser that have been reported are summarized chronologically in a graph (Fig. 1). While the type of laser with more reports is not necessarily more effective, the graph is considered to reflect tendencies of laser types that are established and gain favorable appraisal or no longer in use.

Since dye laser used for the treatment of port wine stain (wavelength: 595 nm) uses hemoglobin as the observer/heater, photothermal conversion occurs efficiently in the blood vessel, and the thermal energy reaches endothelial cells [105]. However, its optical penetration depth is shallow, being about 1 mm in both the skin and mucosa [105]. However, in Nd:YAG laser with a longer wavelength (1064 nm), the optical penetration depth is about 3 mm in the skin and about 6 mm in the mucosa [105]. Although Nd:YAG laser is advantageous compared with dye laser for the treatment of deep lesions, heat is generated also in perivascular tissues, because light is converted to heat as it is absorbed by water contained in the skin and mucosa.

The target of laser treatment for VM is the endothelium of morbidly dilated blood vessels. There is no light that is specifically absorbed by endothelial cells and emits heat. Satisfactory therapeutic results cannot be expected unless treatment is performed by selecting the laser type and modifying the irradiation method based on the understanding of such principles and limitations of phototherapy.

Concerning small VM of the mucosa, tongue, lips, and glans penis, in which scar formation after treatment poses no serious problem, there are a number of reports [106–108] that lesions were resolved by treatment using Nd:YAG laser. There have also been cases in which favorable results were obtained by treatment of anemia due to gastrointestinal bleeding [109] and of symptoms, such as airway obstruction, due to the mass effect of the lesion [110]. While transient purpura and swelling after treatment are unavoidable, they often resolve rapidly [99]. Modifications of the irradiation setting and method are necessary to obtain satisfactory results and avoid serious complications, such as peroneal neuropathy [100] and pigmentation and scar formation of the facial skin [106, 108], and we must learn from the experience of experts.

Nd:YAG laser irradiation by insertion into the lesion under ultrasound guidance has begun to be performed as a treatment to avoid damage of important organs and nerves [98–100], therapeutic experience using this technique has been accumulated, and detailed records and reports have been presented. At present, the results have been satisfactory in terms of safety and efficacy, and standardization of the procedure is anticipated.


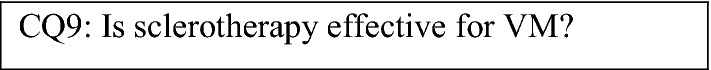


Recommendation:

Sclerotherapy for VM is effective for alleviating symptoms and reducing the size of the lesion and is recommended.

Strength of recommendation: 2 (weak)
Evidence: D (very weak)

#### Comments

VM is lesion that used to be called cavernous hemangioma or intramuscular hemangioma and differ from infantile hemangioma. VM poses problems, such as pain, swelling, and functional impairment, and has been treated conventionally by surgical resection. In Western countries, percutaneous sclerotherapy has a long history. In 1989, Yakes et al. [111] reported ethanol sclerotherapy for VM, and the treatment has since been performed worldwide. Recently, sclerotherapy, which is mildly invasive, permits functional and morphological preservation, and can be performed repeatedly, is widely used. However, as of 2016, sclerotherapy is not covered by medical insurance in Japan. In addition, there has been no randomized controlled trial (RCT) on the usefulness of sclerotherapy for VM compared with surgery or placebo.

As a result of secondary screening, 76, 3, and 3 papers were extracted from PubMed, Cochrane, and JCRM, respectively. They include 3 semi-RCTs, but randomization and blinding were insufficient, and their quality as an RCT was low. Also, the theme evaluated by all these RCTs was “comparison of sclerosing agents in sclerotherapy”, and none compared sclerotherapy with other treatments. Therefore, control groups related to this CQ were not established, and their contribution as a whole is weak. The other literature was all case reports or case series, and the evidence level is D (very weak). As mentioned above, while the evidence level is low, most of the studies reported alleviation of symptoms and regression of lesions in a high percentage (70–90%) of the patients, suggesting the usefulness of sclerotherapy.

The sclerosing agents used included absolute ethanol, polidocanol, ethanolamine oleate, sodium tetradecyl sulfate (STS), and bleomycin. Polidocanol is approved as a sclerosing agent for lower limb varices and esophageal varices, and ethanolamine oleate as a sclerosing agent for esophageal varices. STS is not marketed in Japan. Each sclerosing agent has characteristic complications. Recently, injection of polidocanol, STS, as a foam by mixing with CO_2_ or air has been increasingly performed. Sclerotherapy using ethanol is often performed under general anesthesia, but sclerotherapy using polidocanol or ethanolamine oleate can be performed under local anesthesia.

Three RCTs have been reported as studies that evaluated differences in therapeutic effect according to the sclerosing agent. However, randomization and blinding are insufficient, and their quality as an RCT is low. In addition, the theme evaluated in these RCTs was “comparison of sclerosing agents in sclerotherapy” rather than comparison with other treatments.

Although the evidence level is low, there have been a few case series that reported the usefulness of sclerotherapy, and a wide variety of sclerosing agents including ethanol, polidocanol, ethanolamine oleate, STS, and bleomycin were used. Among studies with a relatively large number of patients, there is a report [112] that sclerotherapy using ethanol in 87 patients with craniofacial VM resulted in a ≥ 75% decrease in size in 23 (32%) and a 25–75% decrease in size in 37 (52%). The results of sclerotherapy using polidocanol in 50 patients with VM were excellent in 19, good in 16, moderate improvement in 13, and unchanged or worse in 2 [92]. The results of sclerotherapy using ethanolamine oleate performed in 83 patients, who were mostly children, were complete remission of symptoms in 79 lesions and significant alleviation in 6 lesions [113]. Sclerotherapy using STS resulted in subjective improvements in 174 (85.3%) of 204 patients [114]. The results of sclerotherapy using bleomycin were complete cure in 185 of 260 patients, marked improvement in 44, and some improvement or no change in 31 [115]. In addition, regarding the size of the lesion, a very satisfactory decrease was achieved in 104, and a satisfactory decrease was achieved in 10, of 120 patients [116].

Papers that evaluated the types of VM that are likely to respond to sclerotherapy include those by Goyal et al. [117], Yun et al. [118], Mimura et al. [91], Rautio et al. [119], Lee et al. [112], Yamaki et al. [120], and Nagao et al. [121] Types of lesions that were likely to be sclerosed were reported to be well-defined small (≤ 5 cm) lesions by Goyal et al. [117], females, lesions showing no or delayed delineation of the draining vein, and lesions well-defined on MRI by Yun et al. [118], small lesions, well-defined lesions, and lesions that show prolonged drug retention by Mimura et al. [91], localized lesions by Lee et al. [112] and Yamaki et al. [120], and slow flow type lesions by Nagao et al. [121] Nomura et al. [122] evaluated the therapeutic effect according to the degrees of functional and gross improvements and reported that the therapeutic effect was greater in head and neck and trunk lesions than in the upper or lower limb lesions. Moreover, Rautio et al. [119] reported that the treatment-related improvement in the quality of life was higher when the lesion did not involve the muscle or was ≤ 5 cm in size.

Various complications ranging from mild complications, such as transient neuropathy and local inflammation, to serious ones, such as myopathy, skin necrosis, and deep venous thrombosis/pulmonary embolism, have been reported. In sclerotherapy using ethanol or polidocanol, particularly serious life-threatening complications have been reported. Qiu et al. [123] reviewed the literature concerning sclerotherapy for VM and reported that shock and pulmonary embolism occurred in 0.19% each of 522 patients who underwent sclerotherapy using ethanol and that ethanol was used at 1 mL/kg in those who developed shock. They also reported that a decrease in blood pressure/bradycardia was noted in 0.61% of 163 patients who underwent sclerotherapy using polidocanol but that its differentiation from vagus nerve reflex was clinically difficult. Wong et al. [124] reported a case of shock after sclerotherapy using 0.86 g/kg ethanol but could be saved. Tachibana et al. [125] reported that 2 patients (1.1%) developed pulmonary embolism and that the amounts of ethanol used were 0.71 and 0.16 mL/kg. Concerning sclerotherapy using polidocanol, also, cardiac arrest in children has been reported by authors including Marrocco-Trischitta et al. [126] and Shimo et al. [127], who used 4 mL of 1% polidocanol (body weight: 20 kg) and 10 mL of 3% polidocanol (15.6 kg), respectively.

In conclusion, sclerotherapy is generally considered effective for VM, but its problems are that the evidence level is low and that the procedure has not been standardized. In addition, serious complications that are rare but life-threatening have been reported, and caution is needed in deciding the dose of the sclerosing agent.


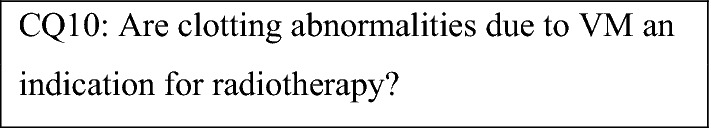


Recommendation:

Radiotherapy should not be performed without careful evaluation because malignant neoplasm, growth disorders, and functional impairment have been reported as late complications.

Many reports included both VM and vascular tumors in the subjects, which make it difficult to assess the therapeutic effects of radiotherapy.

Strength of recommendation: 2 (weak)
Evidence: D (very weak)

#### Comments

As a result of primary screening, 6 and 2 documents were retrieved from PubMed and JCRM. However, as a result of secondary screening, liver hemangioma was excluded, and 10 papers including the references from the previous guideline were reviewed. The reviewed papers were case series or case reports, and the evidence level of the literature as a whole is D “very weak”.

While there have been reports that radiotherapy was performed for the treatment of vascular tumors and vascular malformations, it is difficult to judge whether the treatment was performed by determining the disorders.

According to many reports [128–132], radiotherapy has been performed to treat Kasabach–Merritt phenomenon. However, while there is no mention about Kasabach–Merritt phenomenon, there is a report [133] of 5 cases in which giant hemangiomas accompanied by clotting disorders, thrombocytopenia, heart failure, and bleeding were controlled by multidisciplinary treatment including radiotherapy.

Vascular tumors that cause Kasabach–Merritt phenomenon are considered to be kaposiform hemangioendothelioma or tufted angioma rather than infantile hemangioma [134]. Because VM and infantile hemangiomas are included in other vascular tumors in the lesions described in these reports, they are not considered to be indications of radiotherapy for VM or infantile hemangiomas.

Schild et al. [128] reported 13 cases of symptomatic hemangioma (11 of which were pathologically diagnosed as cavernous hemangioma, but as the report is old, vascular tumors and vascular malformations were not distinguished and were probably included). Radiotherapy at 6.25–40 Gy was carried out in these 13 cases. The lesions were located in the limbs in 5, face in 2, vertebral bodies in 3, pituitary fossa in 1, sacrum in 1, and bladder in 1. Note that organs that should be excluded in this CQ were included.

Of these patients, 2 (1 each with a limb and facial lesion) exhibited Kasabach–Merritt phenomenon and showed normalization of clotting disorder (evaluated according to the platelet count and fibrinogen level) after treatment. However, they were aged 3 years and 5 months, and the lesions may not have been VM.

When the subjects were limited to patients with limb or facial lesions, CR was observed in 2, PR in 4, and no response in 1 in terms of decrease in the lesion size, and CR was observed in 4, PR in 1, and no response in 2 in terms of the control of symptoms.

A serious treatment-related complication, which was unilateral visual impairment, was noted in 1 (14 Gy/8 fr) [128]. These problems have been recognized as late complications of radiotherapy for vascular tumors or vascular malformations; malignant neoplasms, such as breast cancer [135], thyroid cancer [136], and vascular sarcoma [137], visual impairment mentioned above [128], shortening of the lower limb, and restriction of the joint motion range [131].

According to Coldwell et al. [137], late complications of radiotherapy for hemangiomas in infancy include bruise and Stewart–Treves syndrome after the patients reach adulthood. Angiosarcoma is also observed. They reported that the median survival period was 24 months, and the 5-year survival rate was about 10%, in those who developed angiosarcoma.

As observed above, the diagnosis was not confirmed in the reports that have suggested the effectiveness of radiotherapy, and its indications have not been specified. In addition, there have been a considerable number of reports of late complications due to radiotherapy. Thus radiotherapy should not be performed without careful evaluation.


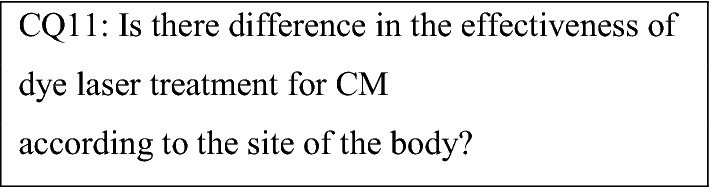


Recommendation:

Dye laser treatment for CM is likely more effective in the face and neck region compared with other sites, and it is more likely to cause complications such as pigmentation in the limbs.

Strength of recommendation: 2 (weak)
Evidence: C (weak)

#### Comments

As a result of literature searches, 176 papers consisting of 139 from PubMed and 37 from JCRM were extracted. They included a few reports that were allegedly RCTs but were not actual RCTs. Therefore, a total of 26 papers consisting of 15 from PubMed and 11 from JCRM including case series with a large number (≥ 100) of relevant cases were selected by secondary screening. In addition, a total of 17 papers were adopted as references for the comments in the guidelines by adding 3 papers in English extracted by manual search to 6 from PubMed and 8 from JCRM considered to be relevant or closely related to the CQ among those selected by secondary screening. Since there was no RCT, the evidence as a whole was rated as C (weak).

Concerning the effect of dye laser treatment for CM, most of the reports were about the effects for hemangioma simplex or port-wine hemangioma in Japan and port-wine stain abroad.

There have been a few papers [138–152] that evaluated the therapeutic results of dye laser treatment according to the site in a small to relatively large number of patients. The laser equipment used varies from early dye laser to pulsed dye laser with adjustable pulse duration with a cooling system, and reports limited to variable-pulse pulsed-dye laser with a cooling system, which is widely used today, are extremely few.

According to many reports [138–149], the response rate is higher in the face and neck region than in the trunk and limbs. In the face, it has been reported that the response rate is higher in the palpebral, forehead and temporal, and lateral buccal regions but is significantly lower in the territory of the 2nd division of the trigeminal nerve (dermatome V2), and that the number of irradiations tends to increase in the midline region, frequently resulting in persistence of redness [150]. There is a report [151] that the response rate did not differ significantly among regions in the lower limb. While the number of patients was small, it has been reported that treatment of the foot involves stronger pain but was less effective than in the face but that the degree of patient satisfaction was relatively high [152].

The incidence of complications of dye laser (bleb formation, depigmentation, pigmentation, scar formation) is reported to be low, being 1.7% in adults, 0.6% in children, and about 1.4% in all patients even when all sites of the body are included, and no significant difference has been reported in the age at the beginning of treatment, Fitzpatrick skin type [153], site, number of treatments, or irradiation energy between those who developed complications and those who did not, but complications may occur more frequently in the lower limbs [154]. Moreover, there is a report [152] that complications, such as pigmentation, depigmentation, and atrophic scar, were observed more frequently in the lower limbs.


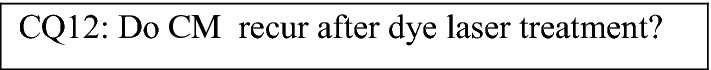


Recommendation:

Although the effectiveness of dye laser treatment for capillary malformations is established, the recurrence rate may increase with time after treatment.

Strength of recommendation: 2 (weak)
Evidence: C (weak)

#### Comments

As a result of literature searches, a total of 211 papers consisting of 149 from PubMed, 53 from Cochrane, and 9 from JCRM were retrieved. They did not include RCTs, and a total of 30 papers consisting of 23 from PubMed and 7 from Cochrane, which were mostly case reports and case series studies, were extracted by secondary screening. In addition, a total of 10 papers that were relevant and closely related to the CQ (including 8 case series) consisting of 7 from PubMed, 2 from Cochrane, and 1 in English retrieved by manual search were adopted as references for the guidelines. Since there was no RCT, the strength of evidence of the group of literature concerning this CQ is C “weak”.

Concerning papers that referred to “whether CM recur after dye laser treatment”, there are 4 retrospective studies [155–158] after treatment by pulsed dye laser (wavelength: 585 nm) with a cooling system, and the recurrence rate was 15.9–35%. Also, there is a report [155] that the recurrence rate increased with time after treatment and was 3.1% after 1 year, 20.8% after 2 years, 40% after 3 years, and 50% after 4 years. Therefore, it is necessary to treat CM with the recurrence after dye laser treatment in mind.

It is difficult to strictly distinguish whether the recurrence is generation of new dilated vessels after laser therapy or it is regeneration of blood vessels damaged due to treatment or re-proliferation of remaining vessels. However, there have been reports that, in an experiment using mice, angiogenesis occurred in the process of wound healing at the site of irradiation in early recurrence [159] and that, in an experiment using hamsters, complete treatment was difficult, and morbid vessels persisted, because coagulation was difficult to induce by dye laser irradiation in vessels ≤ 2–16 μm in diameter [160]. While there is a report [161] that genes affected by dye laser therapy early after treatment were identified, further evaluation is necessary to clarify their relationships with the recurrence.

Concerning the prevention of recurrence, there is a report [162] that the recurrence-free period was long in the patients treated with a variable-pulse pulsed-dye laser with a cooling system (wavelength: 595 nm), which is widely used today, and they were treated within 6 months after birth. In addition, there have been reports of animal experiments using Rapamycin, which inhibits angiogenesis after laser irradiation [159, 163], and of prospective RCT using imiquimod [164, 165], and these treatments were considered effective for the prevention of recurrence. However, careful evaluation by large-scale investigations, including the assessment of the safety concerning drugs, is considered necessary.


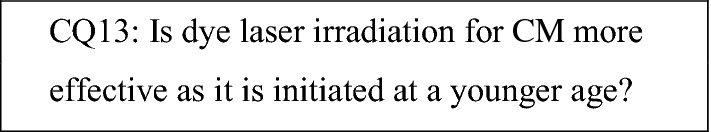


Recommendation:

Laser therapy before the age of 1 year may be effective, and the earliest possible initiation of treatment is recommended as an option.

Strength of recommendation: 2 (weak)
Evidence: D (very weak)

#### Comments

Concerning the timing of treatment for CM, there is the opinion that early initiation of treatment is recommended, because, in young children, the skin is thinner, so the depth of penetration is larger, the vascular wall is also immature, cure after laser irradiation is better, pigmentation is less, and the irradiation area is small, so the treatment efficiency is higher. However, there is still controversy. As a result of secondary screening of past reports, 6 and 1 were extracted from PubMed and JCRM, respectively. While the papers selected by these screening procedures include 2 papers on prospective studies as described below, their conclusions differed, and the evidence level is considered to decline when these references are reviewed together.

Oguri et al. [138] performed a non-randomized controlled trial by dividing children into those aged 0–12 months, 13–24 months, and 25–36 months and observed significant differences in the response rate combining ‘markedly effective’ and ‘effective’ among the groups. They also compared the response rate according to the age in months at the beginning of treatment in the 0-year-old group and reported that the response rate was higher as the treatment was initiated earlier. Furthermore, Nguyen et al. [166] divided their patients into those aged less than 1 year, those aged 1–6 years, and those aged 6 or more years and investigated the correlation between treatment response and age. They reported that those aged less than 1 year and lesions with a size of less than 20 cm^2^ located in the center of the face showed the best treatment response.

Among reports suggesting no difference in the therapeutic effect according to the age at the beginning of treatment, van der Horst et al. [167] studied 100 patients with untreated CM of the head and neck region prospectively and concluded from the results of colorimetry and clinical evaluation that there was no significant difference in the therapeutic effect of pulsed dye laser among the 4 groups in which the treatment was started at the age of 0–5, 6–11, 12–17, and 18–31 years. In the retrospective study of Katugampola et al. [140], also, comparison of 4 groups in which treatment was started at the age of 0–5, 6–12, 13–50, and 50 + years showed no significant difference in the therapeutic effect.

Among the above reports, those did not affirm the usefulness of early laser treatment were relatively old. Also, reports of Oguri et al. [138] and Nguyen et al. [166] indicated laser therapy may be more effective in those aged less than 1 year. In addition, the effectiveness of laser clearly declines when the lesion is elevated or thickens with time. In consideration of “benefits” of early laser treatment and “harms”, which include the occasional necessity of general anesthesia for laser treatment around the eye in small children, the recommendation level was rated as 2D based on the consensus of this guidelines drafting committee.


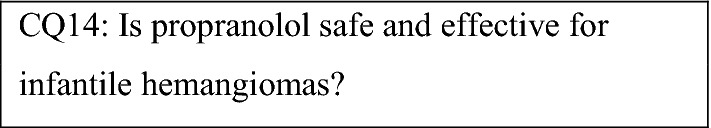


Recommendation:

If administered under careful monitoring, oral propranolol therapy may be the first choice for the treatment of infantile hemangioma.

Strength of recommendation: 1 (strong)
Evidence: A (strong)

#### Comments

(1) Effectiveness: There was the serendipity that regression of hemangioma was induced in a child under steroid therapy with a giant infantile hemangioma by propranolol administered for obstructive hypertrophic cardiomyopathy in 2008 [168]. Based on this report, oral propranolol therapy began to be used for the treatment of infantile hemangioma, and its high efficacy against alarming hemangioma/life-threatening hemangioma in the proliferating phase and in patients with cosmetic problems, such as giant lesions in the face, those with ulcerated and hemorrhagic lesions, and those who may develop functional impairment, has been demonstrated, resulting in its use (Hemangiol^®^, Pierre Fabre Dermatologie, Boulogne, France) as the first choice in Western countries. In addition, its effectiveness for the treatment of hemangiomas after the proliferating phase was also described. Moreover, a group of physicians used propranolol earlier due to cosmetic significance and at the request of the family even in cases of small or localized lesions, and it is also effective in such cases.

A total of 131 papers consisting of 25 from JCRM, 106 from PubMed, and 0 from Cochrane Library were extracted as related to the CQ, “Is propranolol safe and effective for infantile hemangioma?”, and they were subjected to primary and secondary screenings with reduction of hemangioma (effectiveness of propranolol) and treatment-related complications (adverse effects) as outcomes. Twenty-six papers [169–194], most of which were RCTs or observational studies, were adopted.

For example, Hogeling et al. [180] administered placebo or propranolol at 2 mg/kg/day for 6 months with randomization to 40 patients aged 9 weeks–5 years with infantile hemangiomas in the face or sites with the potential for disfigurement. They reported significant improvements in size, redness, and elevation in the propranolol group. Elevated lesions disappeared in 4 of the 19 patients in the propranolol group but none of the 18 patients in the placebo group. As for adverse events, the trial was interrupted in 1 patient due to upper respiratory tract infection, and conditions including bronchiolitis, gastroenteritis, streptococcal infection, cool extremities, dental caries, and sleep disturbance were observed.

Zaher et al. [181] observed 45 patients by randomly dividing them into 15 each treated by oral administration, topical application, and intralesional injection of propranolol. Responses were observed in 60% in the oral group, 20% in the topical ointment group, and 13.3% in the injection group. No major adverse events were noted, and the trial was discontinued in 1 in the oral group and 3 in the injection group due to inconvenience or pain of the treatment.

Malik et al. [182] randomly allotted 30 patients aged 1 week–8 months to propranolol alone, prednisolone alone, or both propranolol and prednisolone. They found that the mean initial response times were lower in the propranolol group than in the prednisolone group but that there was no clear difference between the propranolol + prednisolone group and propranolol alone group. All 10 patients in the propranolol group and 9 patients in the corticosteroid group responded to the 3-month treatment. However, adverse events were observed in 2 of the 10 patients in the propranolol group (asymptomatic hypoglycemia, insomnia) and 9 of the 10 patients in the steroid group (cushingoid appearance, gastrointestinal upset), and were more frequently in the latter group.

Bauman et al. [183] performed a phase 2, investigator-blinded, multi-center RCT in 44 patients aged 2 weeks-6 months. Propranolol or prednisolone (2 mg/kg/day) was administered orally until halted due to toxic effects or clinical response. During 4-months treatment, no significant difference was observed between the two groups, for example, with regression of 5 of the 6 tumors in the corticosteroid group and 9 of the 10 tumors in the propranolol group. For long-term analyses, the effect of prednisolone appeared earlier. While the incidence of adverse events as a whole did not differ between the two groups, severe adverse events were observed in 1 of the 11 patients in the propranolol group but 5 of the 7 patients in the prednisolone group, being significantly more frequently in the latter group.

Léauté-Labrèze et al. [184] carried out an RCT in patients aged less than 4 months by comparing 7 administered and 7 not administered propranolol. Since color changes and softening were observed within 24 h, and the thickness and size of the lesions decreased within 4 weeks in the propranolol group, the treatment was considered useful for the prevention of scarring. No serious adverse effect was observed except asymptomatic mild decreases in heart rate and diastolic blood pressure.

There have also been comparisons between atenolol and propranolol and between laser and laser plus topical propranolol [185, 186]. In 2015, the largest RCT was published in the New England Journal of Medicine, also reporting that propranolol was significantly effective for hemangioma compared with placebo [187]. Hemangioma showed complete or nearly complete resolution after 6-month treatment in 2 (4%) of 55 patients in the placebo group and 61 (60%) of 101 patients in the 3 mg/kg/day propranolol group.

Furthermore, there have also been a few systematic reviews and meta-analyses primarily of observational studies. Menezes et al. [188] reviewed 49 English papers published between June 2008 and September 2010, and summarized 6 studies with 10 or more patients administered propranolol (totally 154 patients). Propranolol was administered to infants with a mean age of 4.5 months at a dose of 2 mg/kg/day in 65% and 3 mg/kg/day in 25.3%. Two-thirds of the patients were treated with propranolol alone. Recurrence was observed in 21% after treatment for a mean of 4.3 months, and adverse events including hypotension, somnolence, wheezing, insomnia, agitation, nightmare, cool hands, night sweat, gastroesophageal reflux disease, and psoriasiform rash appeared in 18.1%.

Marqueling et al. [189] reviewed the therapeutic results in 1264 patients (including 806 girls) in 41 reports published from 2008 to 2012 retrieved from Medline and Cochrane database. The treatment was initiated at a mean age of 6.6 months at 2.1 mg/kg/day and continued for a mean of 6.4 months. The overall response rate was 98%, and the treatment was also effective in clinically problematic areas such as the face (100%), airway (100%), periorbital (98%), head and neck region (97%), and parotid gland (82%). However, recurrence was observed in 17% after treatment. Adverse effects were noted in 371 of 1189 patients. Changes in sleep (136 patients) and acrocyanosis (61) were the most frequent among them, and hypotension was observed in 44, bradycardia in 9, and hypoglycemia in 4 as serious complications. In conclusion, the grade of recommendation was 1, quality of evidence is A, and propranolol was recommended as the first-line drug for complicated infantile hemangiomas. Regarding adverse effects, the grade of recommendation was 1, quality of evidence was A or B. while serious adverse effects may be observed, their frequency is low, and they can be usually avoided by proper monitoring at initiation of treatment.

Xu et al. [190], on the other hand, evaluated volume changes, improvement in overall appearance, visual function, and adverse effects using 15 online databases. The data of 419 cases were analyzed, but meta-analysis was not performed because of the wide differences among studies. Some studies showed superiority of propranolol compared with corticosteroid in reducing volume and improving the overall appearance. No marked difference was noted in adverse effects or visual function.

In addition, in meta-analysis of 16 studies (2629 cases) and 25 studies (795) published in 1965–2012, 69% of the patients responded to 12-month corticosteroid therapy, but the response rate to propranolol was 97% with a significant difference [191].

In periorbital hemangiomas, the response rate to propranolol was found to be significantly higher than that to corticosteroid by meta-analysis of papers published before 2013 [192], and propranolol showed the strongest effect against airway hemangiomas compared with steroid, CO_2_ laser, and vincristine on meta-analysis [193, 194].

From these observations, we concluded that propranolol was significantly more effective than placebo and to be similarly effective compared with corticosteroid. Concerning the safety, propranolol is considered to have significantly fewer adverse effects than corticosteroid. Since there have been several RCTs and systematic reviews or meta-analyses directly related to this CQ, the evidence level is considered to be extremely high.

(2) Meta-analysis: Regarding the effectiveness and adverse effects of propranolol, a large number of systematic reviews and meta-analyses based on observational studies are already present in the above 26 papers. We, therefore, used only 4 reports [180, 182, 183, 187] on interventional studies for meta-analysis.

As a result of meta-analysis, regarding “tumor reduction”, it was found that propranolol had significantly stronger reducing effects than placebo and that it had a stronger reducing effect, which, however, was not significant, compared with corticosteroid. Concerning “complications”, propranolol was compared with steroid and was shown by 2 RCTs to have significantly fewer adverse events than corticosteroid. Since this meta-analysis found significantly stronger reducing effect of propranolol compared with placebo and in fewer complications compared with steroid, and since our results were similar to those of systematic reviews of many existing observational studies considered to have high-quality evidence, we considered that there was a major tendency in this CQ and judged the evidence level as A.

(3) Estimated action mechanism: Beta blockers have a wide range of actions on the blood vessels and vascular endothelium, and have diverse actions on cell proliferation and vascular remodeling. Thus, the action mechanism of propranolol on infantile hemangiomas is still unclear. In vascular endothelial cells, propranolol is considered to induce vascular contraction by suppressing NO production, inhibit renin production, control angiogenesis by regulating the expression of VEGF∙bFGF∙MMP2/MMP9, and induce apoptosis, but it may also affect pericytes and hemangioma stem cells [195–197].

(4) Adverse events associated with propranolol in children

In conducting propranolol therapy, it is necessary to have knowledge about possible adverse effects, their symptoms, and their management. In addition, as there are also preventive measures for, and points of attention about, adverse effects and the timing for discontinuation of propranolol, sufficient explanation to the patients and their families is essential.

Adverse events that have been reported include sleep disorders, peripheral cyanosis, hypotension (symptomatic, asymptomatic), bradycardia (symptomatic, asymptomatic), hypoglycemia, respiratory disorders, gastrointestinal disorders, and mental disorders. Severe cases that require interruption of treatment are few, but particular caution is needed regarding the following points [189, 196–200].

- Since there is the risk of hypoglycemia, the patient should be fed before and after propranolol administration. If the patient cannot be fed, or is vomiting, for some reason, the administration should be suspended.
- Since propranolol has cardiovascular adverse effects, such as hypotension and bradycardia, interviewing for the past history and familial history, examination, and electrocardiography are recommended before treatment. Even if no abnormality is noted on these examinations, hypotension, and bradycardia may occur during treatment. In such cases, interruption of the administration is necessary.
- Propranolol is contraindicated for bronchial asthma as it causes bronchial contraction due to its β2-blocking action. Caution is also necessary in patients who have been suspected to have bronchial asthma.

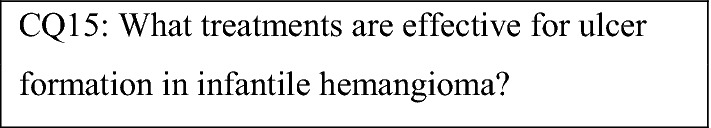

1. Propranolol

Recommendation:

The administration of propranolol is recommended for ulcer formation.

Strength of recommendation: 2 (weak)
Evidence: C (weak)

2. Topical administration of antibiotics

Recommendation:

Topical and systemic administration of antibiotics is recommended for ulcer formation.

Strength of recommendation: 2 (weak)
Evidence: D (very weak)

3. Dressings

Recommendation:

The use of dressings is recommended for ulcer formation.

Strength of recommendation: 2 (weak)
Evidence: D (very weak)

4. Laser therapy

Recommendation:

Although laser therapy may be effective in some patients with ulcer formation, the evidence is not considered sufficient at present.

Strength of recommendation: 2 (weak)
Evidence: D (very weak)

5. Systemic administration of steroid

Recommendation:

Systemic administration of steroid is recommended not to be performed for ulcer formation.

Strength of recommendation: 2 (weak)
Evidence: D (very weak)

6. Platelet-derived growth factor preparations

Recommendation:

The accumulation of cases is insufficient for the judgment of the recommendability of the use of platelet-derived growth factor preparations for ulcer formation.

Strength of recommendation: No recommendation
Evidence: D (very weak)

#### Comments

Concerning this CQ, 42 papers in Japanese and 156 in English were retrieved. As a result of their primary screening, 47 papers were submitted to secondary screening for this CQ. None of them were about studies with a high level of evidence, such as RCT, and they were all retrospective studies, case series, or case reports.

As a result, 15 papers in English were adopted, and the evidence level was C for propranolol alone, because of the presence of a prospective controlled trial, but D for other treatments, because the related papers were case reports or case series.

According to cross-sectional analysis in a multicenter prospective cohort study in 1096 cases of infantile hemangioma by Chamlin et al. [201], it was complicated by ulcer, which was or was not bleeding, in 173 (15.8%), the median age of the patients was 4.0 months (SD = 8.5, mean = 6.6 months), and the age at the first examination was significantly lower in patients with ulcerated hemangioma (median = 3.5 months, mean = 3.98 months) than in those with non-ulcerated hemangioma.

By the site, ulcer formation was observed in 21 (30%) of 71 patients in the lower lip, 25 (25%) of 100 patients in the neck, and 46 (50%) of 93 patients in the perianal/perigenital area, and the frequency was statistically lowest in the upper eyelid (p = 0.0140).

Ulcer formation was observed more frequently in mixed or segmental hemangiomas. Bleeding was noted in 78 lesions (41%) and was mild in 56 (29%), moderate in 11 (6%), and severe in 4 (2%). Severe bleeding occurred in 3 lesions in the limbs and 1 lesion in the face, and bleeding occurred in 2 cases at home. Two cases required blood transfusion by hospitalization, because they showed symptoms due to serious bleeding. Of the ulcerated hemangiomas, 67 (35%) were in the proliferating phase.

Ulcerated hemangiomas required treatment (odds ratio (OR) = 6.86, 95% CI = 3.70–12.71, p < 0.0001), and non-ulcerated hemangiomas were observed (OR = 19.01, 95% CI = 11.23–28.88, p < 0.0001). Ulcerated hemangiomas tended to be treated by conventional wound care and pulsed dye lase (OR = 2.03, 95% CI = 1.19–3.46, p < 0.0091), and non-ulcerated hemangiomas were treated by topical glucocorticoid administration (OR = 2.57, 95% CI = 1.49–4.43, p < 0.0007) and surgical resection (OR = 2.04, 95% CI = 1.08–3.86, p < 0.0286).

However, propranolol has recently been suggested to be effective regardless of the presence or absence of ulcer formation, and as it has few adverse effects, it is expected to become the first choice treatment in the future.

[Treatments]

1. Oral propranolol

Hermans et al. [172] treated 20 previously treated patients with ulcerated infantile hemangioma using propranolol and compared them with 36 patients treated without propranolol. The administration was initiated by hospitalization, and the dose was increased from 0.7–1.0 to 2.0–2.5 mg/kg/day in 3 divided doses at an interval of at least 3 days. The blood pressure, heart rate, and blood sugar level were monitored during the initial administration period, and the administration was continued on an outpatient basis until the age of 1 year. The mean age at the beginning of propranolol administration was 3.5 months, and the mean duration of administration was 9.1 months. Not only the color and elevation of the lesion but also pain was reduced from early after the beginning of administration. The administration was concluded before the age of 1 year in 19 patients, and no recurrence of ulcer was noted in any of these patients except that some reactivation (enlargement) of hemangioma was observed after the discontinuation in 4 of these patients.

The mean time until complete cure of ulcer was 8.7 weeks, and those in whom the administration was initiated later (> 3.5 months) tended to require a longer time until cure than those in whom the administration was initiated earlier (p = 0.025). Also, analysis using the *t* test showed a significant difference in the time until disappearance of the tumor, which was 8.7 and 22.4 weeks (t = 2.6, df = 38, p = 0.012, 95% CI = 3.2-24.2) in the treated and control groups, respectively. Temporary sleepiness/malaise was observed in 6 patients, irritable before falling asleep in 2 patients, coldness of the limbs in 6 patients, anorexia in 2 patients, and gastrointestinal disorders (diarrhea, vomiting) in 1 patient, but no adverse event was noted in 9 patients.

Vercellino et al. [202], who started the administration at 1 mg/kg/day and increased to 2 mg/kg/day, and Sadykov et al. [203], who started the administration at 2 mg/kg/day, also reported that propranolol was effective.

2. Topical and/or systemic administration of antibiotics

Kim et al. [204] topically administered antibiotics in 40 patients with ulcerated hemangioma and reported that the results were better in 37 patients (92.5%), worse in none, and no change in 3 patients (7.5%). They also systemically administered antibiotics in 26 patients and reported that the results were better in 24 patients (92.3%), worse in 2 patients (7.7%), and no change in 0 patient.

Wananukul et al. [205] topically and/or systemically administered antibiotics in 41 patients with ulcerated hemangioma and reported improvement in 19 patients (46%).

Pandey et al. [206] treated 608 patients showing ulcer formation with an ointment containing an antibiotic (mupirocin, sodium fusidate, sisomicin, or metronidazole) combined with systemic administration of an antibiotic (amoxiclav at 20–40 mg/kg/day) in those with ulcers with an area of > 10 cm^2^ and examined the effectiveness of treatment according to the time until cure. The time until cure was 32.63 ± 13.06 days in superficial lesions, 42.89 ± 19.89 days in mixed lesions, and 57.03 ± 16.12 days in extensive lesions, with a mean of 40.09 ± 19.41 days in all lesions combined, showing significant differences among the 3 groups (p < 0.05). They also reported that the time until cure was significantly longer in larger (> 10 cm^2^) than smaller ulcers (p < 0.05).

3. Dressings

Kim et al. [204] treated 25 patients using dressings and reported that the results were better in 23 patients (92%), and no change in 2 patients (8%). Oranje et al. [207] applied polyurethane film and reported rapid relief of pain and cure of ulcer in 1–2 months. In addition, Bauland et al. [208] treated 41 patients using a non-adhering dressing containing an antibiotic and reported that the results were good in 26 patients (63.4%), moderate in 5 patients (12.2%), and little change in 10 patients (24.4%).

4. Laser therapy

In the 1980–1990s, there were reports [209, 210] of argon, Nd YAG, and KTP, but recent reports [211, 212] are primarily about treatment using dye laser. Morelli et al. [209] treated 37 patients with ulcerated hemangioma by dye laser irradiation (SPTL1b^®^ (Candela Corporation, Wyland, MA, USA), wavelength: 585 nm, spot size: 5–7 mm, irradiation power: 5–6.8 J/cm^2^, pulse width: 0.45 ms) and reported that the number of irradiations until cure was 1 in 26 patients (68%) and 2 in 8 patients (21%) and that the mean period from the first treatment until cure of ulcer was 2.84 ± 0.22 weeks. Lacour et al. [210] irradiated 8 patients with ulcerated hemangioma that resisted conventional treatments using the same equipment and reported acceleration of cure. David et al. [211] performed dye laser irradiation (PhotoGenica V^®^ (Cynosure, Westford, MA, USA) , wavelength: 585 nm, spot size: 5–7 mm, irradiation power: 5–6.8 J/cm^2^, pulse width: 0.3–0.5 ms) in 78 patients and reported the effectiveness of laser therapy alone in 72 (92.3%). Also, Michel [212] performed 1 or 2 irradiations using Dermobeam 2000^®^ (Deka MELA, Calenzano, Italy) with a cooling system 595 nm (2 pulsed irradiations with a 10% overlap, spot size: 7 mm, irradiation power: 4–8 J/cm^2^) and reported resolution of pain in 10 of the 12 patients. Moreover, Di Maio et al. [213] performed laser treatment in 65 patients with hemangioma with ulcer and reported that the effect was excellent and that no clear adverse events were observed, because scarring, which was noted in a few patients, did not differ markedly compared with scarring that occurs after conventional treatments.

However, Kim et al. [204] treated 22 patients with pulsed dye laser and reported that the results were better in 11 patients (50%), worse in 1 patient (4.5%), and no change in 4 patients (18.2%), but warned that 5 patients in the proliferating phase showed ulcer formation after irradiation.

As observed above, although there have been several reports of the effectiveness of laser therapy against ulcer as factors of “benefit”, many reports are relatively old and lack controls, and the evidence is not considered sufficient. Further accumulation of cases is necessary. Laser may be effective in limited patients, but as there is the risk of ulcer formation as an adverse effect of laser irradiation of infantile non-ulcerated hemangioma, greater caution is needed in treating already ulcerated lesions.

5. Steroids

There have been few reports on steroid therapy focusing on ulcer. Kim et al. [204] treated 7 patients by local steroid injections and reported that the results were better in 4 patients (57.1%), worse in 1 patient (14.3%), and no change in 1 patient (14.3%). They also systemically administered steroid to 22 patients and reported that the results were better in 16 patients (72.7%), worse in 1 patient (4.5%), and no change in 5 patients (22.7%). Based on these results, they considered that the treatment was effective for reducing the lesion size, and there are few other reports suggesting the effectiveness of steroid. Considering that the patients are infants and that there are other treatment options, steroid cannot be recommended at present.

6. Topical preparations of recombinant human platelet-derived growth factor

0.01% becaplermin (Regranex^®^ (Ortho-McNeil Pharmaceutical, Raritan, NJ, USA) ) is a preparation for diabetic foot ulcer approved by the FDA in 1997. Sugarman et al. [214] and Metz et al. [215] reported its effectiveness for the treatment of ulcerated hemangioma in 1 and 8 patients, respectively, but its effectiveness cannot be appraised at present because of the small number of cases.


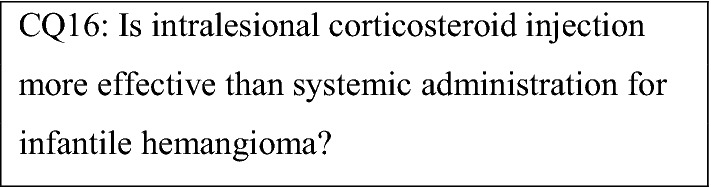


Recommendation:

Treatment using corticosteroid is effective for inducing early regression of hemangioma. While no significant difference is observed in the effectiveness between intralesional injection and systemic administration, attention to complications including those at the administration site, such as the periocular region, on local injection and those, such as hypertension and growth retardation, on systemic administration is necessary.

Strength of recommendation: 2 (wean)
Evidence: B (moderate)

#### Comments

As a result of primary screening, 99, 9, and 35 papers were extracted from PubMed, Cochrane, and JCRM, respectively, and 4 papers in English were subjected to secondary screening for this CQ. There was 1 report of an RCT, but the other reports were about case series while they evaluated a large number of cases. In addition, 2 papers on complications considered important in relation to intralesional corticosteroid injection for periocular lesions were added by manual search. Since there is a report of an RCT, and since other case series studies with a large number of subjects presented the results that there was no significant difference in the effectiveness of corticosteroid depending on the administration method, the strength of evidence was rated as “B”.

There was 1 report [216] of an RCT focusing on “Is intralesional corticosteroid injection more effective than systemic administration for infantile hemangioma?”. In this trial, the subjects were divided into control, oral administration (prednisolone at 2 mg/kg/day every other day for 6 weeks), and intralesional injection (triamcinolone at 1-5 mg/kg with a maximum of 30 mg once a month for 6 months) groups, and the lesion size was significantly reduced in the treated groups compared with the control group. While no significant difference was noted between the oral administration and the intralesional injection groups, the reduction rate tended to be larger in the local injection group, and local injection was concluded to be slightly superior [216].

There were reports [217, 218] of case series with more than 1000 subjects, but the findings were not statistically analyzed. Although both intralesional injection and oral administration were effective, there was also a mixed group of intralesional injection and oral administration, the condition of patients varied among the 3 groups (intralesional injection, oral administration, mixed), and the effectiveness according to the administration method was not shown. Regarding complications, systemic symptoms, such as hypertension, retarded body weight gains, and cushingoid appearance, were reported to be more frequent on oral administration than intralesional injection [217, 218]. Moreover, concerning complications, in one report [219], periocular lesions were excluded from the targets of intralesional injection to avoid its effect on visual function. Indeed, there have also been case reports [220, 221] that visual impairment was caused by occlusion of the retinal artery after intralesional corticosteroid injection for periocular hemangiomas. Currently, in Japan, intralesional corticosteroid injection is a treatment unapproved by the national health insurance system.


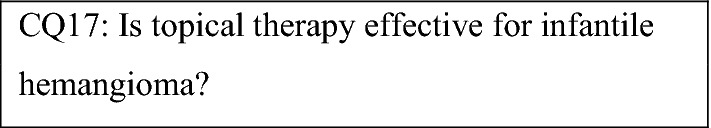


Recommendation:

Although it must be noted that there are no reports of comparison with placebo and that the degree of improvement is smaller compared with systemically administered drugs, topical medication can be an option for the treatment of infantile hemangioma with no risk of complications if drugs with milder adverse effects are selected.

Strength of recommendation: 2 (weak)
Evidence: C (weak)

#### Comments

As a result of literature searches, a total of 111 papers consisting of 70, 7, and 34 papers from PubMed, Cochrane, and JCRM, respectively, were extracted. They included 1 RCT study. Including this RCT, 48 papers were extracted by secondary screening. In addition to the papers selected by secondary screening as closely related to the CQ, a total of 47 papers obtained by manual search were adopted as reference for the preparation of guidelines. There was 1 RCT, and comparative studies of therapeutic results by topical therapy and case series studies with a relatively large number of subjects were adopted as papers of relatively high quality, the strength of evidence was rated as C “weak”.

In the reports related to the CQ:

(1) Drug type: The drugs were classified into imiquimod, timolol, propranolol, corticosteroid, and others [215, 222–225].

(2) Drug concentration and dosage form: Imiquimod was used as a 5% cream [222, 226–233], timolol as 0.5% ophthalmic solution or gel [222, 223, 232, 234–243], propranolol as 1% ointment [181, 234, 241, 244, 245], and corticosteroid were often used as ointments of agents ranked as relatively strong such as clobetasol propionate, halobetasol propionate, and betamethasone dipropionate [246, 247].

(3) Methods for topical application: Frequent administration methods were once a day every other day for imiquimod, 2 times a day every day at 1–2 drops each time for timolol, 2 times a day every day for propranolol, and 2 times a day every day for corticosteroid.

(4) Methods for efficacy evaluation: Comparison of gross findings and photographs were adopted in all papers. The area was compared using photographs in one report [227]. There was also a report [228] of half-side test for a control.

(5) Adverse effects: No systemic adverse effect was reported, and most adverse effects were local. Imiquimod caused pain, flare, and erosion relatively frequently [231, 233]. Few local adverse effects were reported for timolol and propranolol [232, 234, 241, 242, 245]. No local adverse effects were reported also by corticosteroid [246, 247].

(6) Relative advantages of drugs Imiquimod has been reported to have usefulness comparable to that of topical beta blockers, but it is not considered superior in terms of adverse effects [226, 228, 230, 232].

Corticosteroid was not shown to be superior in efficacy compared with beta blockers.

There was also one RCT study [181] concerning the CQ, which is related to topical propranolol concerning drugs. In this RCT, 15 each of a total of 45 subjects were allotted to oral (propranolol at 2 mg/kg/day, 2 times a day), topical (1% propranolol water soluble ointment, applied 2 times a day), and local injection (1 mg/1 mL, 0.2 mL/1 cm in diameter, 1 mL/injection at the maximum, 1 time/week) groups. Ten patients (66.7%) in the topical group responded, but they were fewer than 13 (86.7%) in the oral group. The time until the appearance of the effect and time until complete cure were also longer in the topical group than in the oral group. Concerning complications, none was observed in the topical group, but 1 patient in the oral group showed unexplained syncope as an adverse effect and was excluded. While decreases in the heart rate and blood pressure were observed in 3 in the oral group, they did not necessitate interruption of the study. In the local injection group, 8 (53.3%) responded, but 3 were lost due to pain. From these results, the study concluded that topical therapy is an option to be evaluated for patients with a risk of adverse reactions to oral medication. While there were no reports of comparison under the same conditions, comparative studies of therapeutic results by topical therapy and case series studies with a relatively large number were adopted as relatively high-quality papers. In all these reports, topical therapy of beta blockers (propranolol, timolol) was effective to an extent with no serious complications.

Thus, topical therapy, particularly, of beta blockers is considered generally useful, but there has not been a report of its comparison with placebo, and further accumulation of cases is necessary.

Research by comparison between dye laser treatment and topical beta blocker therapy is considered to be necessary.


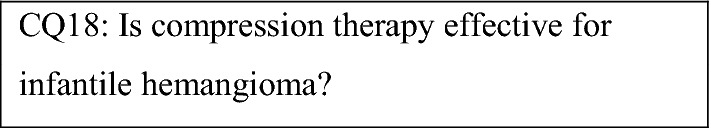


Recommendation:

Although appropriate compression method must be performed for individual patients, compression therapy may be regarded as an option on condition that the therapy is carried out by a skilled physician. Sufficient attention to skin abnormalities and local/neighboring growth disturbance due to the compression are needed.

Strength of recommendation: 2 (weak)
Evidence: D (very weak)

#### Comments

While 23, 1, and 14 papers were extracted from PubMed, Cochrane, and JCRM, respectively, only 3 case reports remained to be reviewed as a result of primary and secondary screening. Thus, the evidence level is very low at D (very weak).

According to a case report of ulcerated infantile hemangiomas of the limbs by Kaplan et al. [248], the ulcers of most patients showed rapid improvements and cured within 2 weeks by compression therapy using the self-adherent wrap Coban^®^ (3M, St. Paul, MN, USA) combined with topical treatment with an antibiotic ointment (or early systemic antibiotic administration when secondary infection was apparent). They concluded that, compared with antibiotic ointment alone, its combination with compression therapy was more effective, and is a safe and easy treatment that promotes regression of hemangiomas.

Ochi et al. [249] reported 12 cases of infantile hemangioma (9 girls and 3 boys with a mean age of 8.4 months; sites of the lesion: limbs in 6, head and neck in 5, and trunk in 1). By treatment using elastic bandages (5 patients), Presnet^®^ (ALCARE, Tokyo, Japan) (4), supporter (1), or Elatex^®^ (ALCARE, Tokyo, Japan) and cryotherapy (2), the hemangiomas disappeared or decreased in size in 11 of the 12 patients, with only 1 (head and neck) showing no improvement. The time until the disappearance of the lesion in the 11 responders was 2 months to 3 years (mean: 19.5 months), no complications associated with compression therapy were noted, and the authors recommended early initiation of compression therapy if the site of the lesion can be compressed.

Totsuka et al. [250] treated 3 girls with parotid gland hemangiomas (mean age: 4.3 months) by splinting using a resin plate and compression using a handmade cap. The mean duration of treatment was 13 months (8–16 months), and the patients were followed up until a mean age of 4.6 years (2–7 years), resulting in clinical and echographic disappearance of hemangioma in all 3. Since infantile hemangiomas often regress spontaneously, it is impossible to conclude that they regressed due to compression therapy, but they reported the therapy to be safe and effective.

Thus, concerning factors related to “benefits” of compression therapy, there are reports that suggest the effectiveness of compression methods appropriate for sites (elastic bandages, Present, splinting with a resin plate). However, it must be noted that they are all old reports. Concerning factors related to “harm”, while compression is a relatively safe and simple method without reports of serious complications, the occurrence of dermatitis and growth disturbance at the site of compression or surrounding areas is considered possible. The recommendation level was set at 2D with consensus of the present guidelines preparation committee on condition that the treatment is performed carefully by a skilled physician in consideration of these points. The present guidelines do not exclude compression therapy, but it is necessary to consider oral propranolol, oral administration or local injection of steroid, and laser therapy first for infantile hemangiomas that need treatment.


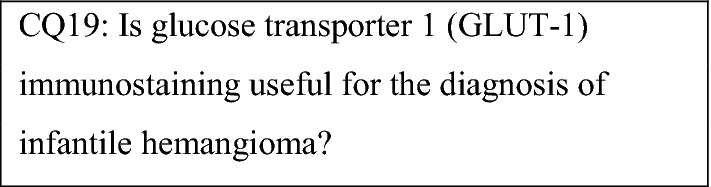


Recommendation:

Immunostaining for GLUT-1 is positive in the proliferating, involuting, and involuted phases, shows high sensitivity and specificity, and is useful for the diagnosis of infantile hemangiomas if the clinical diagnosis is difficult.

Strength of recommendation: 2 (weak)
Evidence: C (weak)

#### Comments

To evaluate whether GLUT-1 immunostaining is useful for the diagnosis of infantile hemangiomas, the literature was searched first for the following key words.

Infantile OR juvenile AND hemangioma AND marker AND immunohistochemistry

The search of JCRM resulted in 26 hits, but none of them performed analysis of GLUT-1 or evaluated its usefulness by comparing infantile hemangioma with other hemangiomas/vascular malformations even if GLUT-1 was analyzed. The search of PubMed resulted in 182 hits. From these papers, those that deserved detailed analysis were selected according to the following criteria.

(1) Those in which GLUT-1 immunostaining was performed for infantile hemangioma or other hemangiomas/vascular malformations.

(2) Those that were retrospective epidemiological studies rather than reports of one case.

Fifteen research papers selected by these criteria were analyzed in detail.

In 7 of these reports [251–257], infantile hemangiomas were stained using GLUT-1 simultaneously with other hemangiomas/vascular malformations, and differences in positive/negative results were evaluated. Of all cases reported in the 7 papers, GLUT-1 was positive in 268 of the 273 cases of infantile hemangioma and negative in 244 of the 247 cases of lesions other than infantile hemangioma. There were also 4 papers [258–261] in which GLUT-1 staining was performed for clinically typical infantile hemangiomas and hemangiomas that need to be differentiated from infantile hemangioma although they were not simultaneously stained in the same paper. When the 4 papers were combined, GLUT-1 was positive in all 8 cases of infantile hemangioma and negative in all 49 cases of non-infantile hemangioma. When the above cases are totaled, GLUT-1 was positive in 276 of the 281 cases of infantile hemangioma and negative in 293 of the 296 cases of non-infantile hemangioma, and the sensitivity and specificity of GLUT-1 positivity for infantile hemangioma were 98.2 and 99.0%, respectively.

The usefulness of GLUT-1 staining has also been confirmed by re-evaluation of cases that were initially examined by hematoxylin–eosin staining alone [262–265]. There have been 4 papers in which cases were re-evaluated using GLUT-1 staining, and 1 paper reported that the diagnosis was impossible by HE staining alone in 18% of the cases [262].


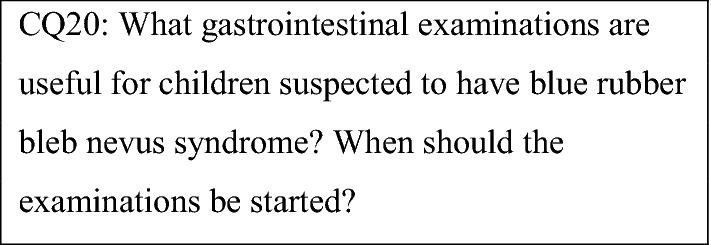


Recommendation:

It is recommended to start screening by examinations including blood tests and fecal occult blood test as early as possible. In children suspected to have gastrointestinal bleeding, the usefulness of endoscopic examination, red blood cell scintigraphy (99mTc-labeled red blood cells), and single photon emission computed tomography-CT (SPECT-CT) has been reported for the identification of the source of bleeding. If no abnormality is detected by screening, and search for gastrointestinal lesions needs to be performed to diagnose this disease or evaluate the future risk of bleeding, there is no standard for its timing. Among the examinations that led to the detection of gastrointestinal lesions in past reports, CT and MRI can be performed with relatively mild invasion and from an early stage.

Strength of recommendation: 2 (weak)
Evidence: D (very weak)

#### Comments

Gastrointestinal lesions of blue rubber bleb nevus syndrome (bean syndrome) are observed in the entire digestive tract, but they frequently appear, particularly, in the small intestine. Since it is an extremely rare disease, the literature is primarily case reports and reviews, and there have been no reports of clinical studies of many cases that are relevant for the CQ. Therefore, we investigated examinations that were useful for the detection of gastrointestinal lesions in reports, primarily, of child cases. Lesions in the small intestine are difficult to observe by conventional endoscopy, but techniques such as double-balloon endoscopy, capsule endoscopy, CT enterography, CT, and MRI as well as upper and lower gastrointestinal endoscopy have been reported to be useful [266–276].

As a result of database searching, 11 papers in English were adopted through primary and secondary screening. All papers selected by these screening processes were case reports or case series, and the strength of evidence is “D (very weak)”.

There is no clear standard as to when the examinations should be initiated. However, neonates who developed gastrointestinal bleeding shortly after birth have been reported [270], and the earliest possible examinations are necessary if this disease is suspected. Invasive examinations are difficult to perform in small children, but blood tests (presence or absence of anemia or consumption coagulopathy) and fecal occult blood tests can be performed. If gastrointestinal bleeding is suspected, procedures such as endoscopy, particularly, double-balloon endoscopy and capsule endoscopy, 99mTc-labeled red blood cell scintigraphy, and 99mTc-labeled red blood cell SPECT-CT have been reported to be useful for the determination of the source of bleeding [266, 268, 271, 275].

If no abnormality has been detected by screening tests, and if search for gastrointestinal lesions needs to be performed non-emergently to diagnose this disease or evaluate the future risk of bleeding, there is no standard for the timing, which may vary among facilities. Among the above examinations, CT and MRI can be performed earlier and with relatively milder invasion, and are worth attempting if this disease is suspected. The necessity of the other examinations for the gastrointestinal lesions mentioned above should be considered when the patient reaches the age that tolerates the examinations.


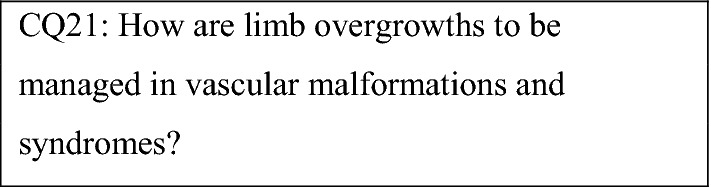


Recommendation:

If leg-length inequality is insignificant, shoe lift is recommended. As significant inequality causes gait disturbance complicated with scoliosis, surgical treatment aimed to arrest epiphyseal growth is performed in the growth period. Shortening of the femur or tibia may be performed as an additional treatment. Bone elongation of the intact side is considered effective for the correction of leg-length inequality.

Strength of recommendation: 2 (weak)
Evidence: D (very weak)

#### Comments

As a result of literature searches, 40 papers in English and 4 papers in Japanese were retrieved by primary screening. Of these papers, 17 in English and 4 in Japanese were extracted by secondary screening. As for the control of overgrowth of limbs, measures against leg-length inequality and soft tissue hypertrophy are separately discussed and regarded as effective, but these papers were all classified either as case reports or as general discussions. Therefore, the evidence level is rated as “very weak”, and the recommendation level as “weak”.

In vascular malformations, typical disorders with hypertrophy of the affected limbs are Klippel–Trenaunay syndrome and Parkes Weber syndrome and most of the papers refer to the management of limb overgrowth due to vascular malformations were written about these disorders. The literature regarding lesions at different sites is commented on below.

### Lower limbs

In most reports, treatment for the overgrowth of the lower limbs were aimed to prevent physical disorders caused by leg-length inequality. Some reports particularly mentioned treatment for foot lesions.

1. Correction of leg-length inequality

If the leg-length inequality is ≤ 2 cm, the management of leg-length difference and accompanying scoliosis is considered possible by the use of shoe lift [277–281]. If the leg-length inequality is ≥ 2 cm, significant gait disturbance, postural abnormalities, and compensatory changes in the contralateral limb are likely to develop, and before consequent unphysiological gait leads to irreversible impairment, surgical treatment to correct the leg-length inequality should be considered [277–281]. Long-leg radiography is useful for determining the best time for surgery [282], and the measurement of the leg-length by long-leg radiography or CT is reported to be effective [278]. Surgical treatment reported in the papers are as follows.

### Treatment for overgrown limbs affected by vascular malformations

Jacob et al. [277] performed epiphysiodesis in 41 patients with a leg-length inequality of ≥ 2 cm among 252 patients with Klippel–Trenaunay syndrome and reported improvement in more than 90% of the patients. The effectiveness of this surgery is also confirmed by other review articles [277–281]. The effectiveness of shortening of the femur and tibia was reported in the review by Capraro et al. [278] The fixation period is considered to be shortened as a whole by simultaneously performing femoral or tibial shortening in addition to epiphysiodesis. Redondo et al. [280] recommended endoscopic growth control of the epiphyseal plate in the distal end of the femur for patients with a leg-length inequality of ≥ 2 cm. Capraro et al. [278] did not recommend growth control with the epiphyseal stapling because of the unpredictability of the results and high frequency of complications. The appropriate time for surgical intervention on affected limbs is reported to be around the age of 11 years [280].

### Elongation of the intact leg

Tanaka et al. [283] performed bone elongation of the intact limbs using an external fixator in adult patients with mild structural scoliosis and reported that the procedure was effective for correcting the leg-length inequality and scoliosis. Jacob et al. [277] also recommended bone elongation of the intact limb using Ilizarov external fixation apparatus in their review.

### Popliteal vein ligation

Servelle [284] hypothesized that elongation of the affected limbs was due to a high venous pressure and performed ligation of the popliteal vein of the intact limb in 48 children, and they reported significant improvement in leg-length inequality. However, there are also negative views, saying its effectiveness is uncertain [278].

2. Foot lesions

Redondo et al. [280] recommended resection of the toes (ray resection) and debulking for wearing shoes and cosmetic improvement. Gates et al. [285] notably reported that compared with ray resection, wound healing of the stumps was poor after major resection.

### Upper limbs

Asymmetry due to hypertrophy of the upper limb less frequently causes impairment of ADL than that of the lower limb. In one article, resection in patients with functional impairment due to marked finger deformities is reported [282], but articles reporting treatment for upper limb overgrowth are very few. While debulking has been reported to be advantageous from the cosmetic viewpoint [279], it has also been reported to induce exacerbate of edema of the affected limb [284], causing complications including cicatricial contracture, recurrence of the lesion, and refractory ulcer [278], and sufficient caution is necessary.


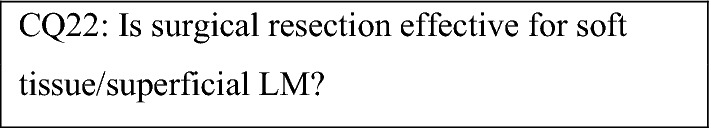


Recommendation:

Although surgical resection is an effective treatment, it should be performed after comprehensive evaluation of cosmetic aspects, prognosis, functional prognosis, resectability, and possibility of recurrence/complications.

Strength of recommendation: 2 (weak)
Evidence: D (very weak)

#### Comments

[Process of preparation of recommendation]

Surgical resection is one of the major treatment options performed for LM. Although LM can be cured by total resection, the objective of treatment is not necessarily total resection, because the disease is not malignant, and surgical resection is often carried out for cosmetic, functional, and symptomatic improvements. Cosmetic problems are considered to be particularly serious if the lesion is located in superficial areas such as the body surface and soft tissue. However, surgical resection has been known to cause complications including hemorrhage, infection, deformation, and nerve paralysis.

In evaluating whether resection is effective, the balance between its positive aspects and negative aspects, such as complications, is important. For soft tissue/superficial LM, in which cosmetic improvement is important, problems including in what situations resection can be performed, whether there are criteria for the selection of resection, and, as there are differences in the incidence of complications, cure rate, and recurrence rate depending on the circumstances, whether its indications should be evaluated under different conditions are unclear. Therefore, the CQ, “Is surgical resection effective for soft tissue/superficial LM effective?”, was formulated, and the current knowledge was summarized.

<Literature search and screening>

As a result of literature search, 105 papers in Japanese and 348 papers in English were subjected to primary screening. Of these papers, 5 in Japanese and 42 in English were subjected to secondary screening concerning this CQ. They did not include papers with a high level of evidence, such as a systematic review and RCT, and all of them were case series or case reports. As a result, in the evaluation of this CQ, the results and discussion in each case series were integrated.

<Review of observational studies (case series)>

The effectiveness of resection of LM was evaluated from the following 5 viewpoints: (1) Effectiveness regarding the prognosis (mortality), (2) resection rate of the lesions (resectability), (3) functional outcome after resection (function), (4) recurrence rate (recurrence), and (5) complications.

### Results of review

Generally, the rate of successful surgical resection is high, and ≥ 90% resection is reported to be possible in 60% or more of the patients [286–288]. This also applies to the head and neck region, which is the frequent site of the lesion [286]. However, the percentage of resectable lesions decreases from the cystic to mixed and to cavernous type [286]. Since many LM are distributed diffusely in the skin and subcutaneous adipose tissue and around structures including muscles, blood vessels, and nerves, resection of the lesion involves resection of normal tissues in varying degrees. In lesions that show complicated distribution in the head and neck region, complications after surgical resection are observed relatively frequently. Serious complications including nerve paralysis, hematoma, local necrosis, sepsis, deformation, salivary fistula, hoarseness, airway obstruction, and malocclusion have been reported [286, 287, 289–297], and facial nerve paralysis is likely to result from resection, particularly, of LM infiltrating the parotid region [289]. By the site, the incidence of complications increases as the area of involvement widens from unilateral to bilateral, from below to above the lingual bone, both sides, and both above and below the lingual bone [293, 296]. Postoperative death may occur in patients with a severe neck lesion, but the extent of the effect of surgical resection is unclear [287, 288, 298]. Postoperative recurrence is closely related to the resectability of the lesion depending on its distribution, and lesions that are difficult to resect due to a wide area of involvement and a strong tendency of infiltration have been reported to be associated with recurrence [296].

### Limitations

Indications for surgical resection vary among papers, and differences in the patient background must be considered in the evaluation of the effectiveness of resection. While there were many reports that surgical resection was performed in combination with sclerotherapy, and resection is considered to have been performed when more favorable results were expected from resection rather than sclerotherapy, criteria for their selection are unclear. Therefore, there is certainly the large bias of individual variation in the circumstances, and it was clearly impossible to conclude that resection is uniformly effective.

<Summary>

While the effectiveness of surgical resection for soft tissue/superficial LM was evaluated, there was no literature with a high level of evidence. One of the major reasons is the diversity of the lesion type (cystic or cavernous), area of involvement, and history of other treatments. Because of this diversity, the condition of patients is considered to show an extremely wide variation, and their generalization is impossible. However, if conditions, such as the type of the lesion (cystic or cavernous), site of origin, and relationship with other treatments are restricted, tendencies were observed in functional prognosis, recurrence rate, and contents and incidence of complications.

While the resection rate of lesions by surgical treatment was suggested to be generally high, selection criteria for resection were unclear. Therefore, it is speculated that resection was performed for patients clinically judged to be treated more effectively by surgery. However, since there were some serious complications of surgical resection that persist as sequelae, their possibility should be evaluated carefully in performing surgical resection. The risk of resection has been suggested to vary with conditions of the lesion. The functional outcome is poor, and the recurrence rate and incidence of complications after resection are high, in those that occupy a wide area and those that are accompanied by symptoms such as airway obstruction.

From these observations, we propose at present, “While surgical resection is often effective, it must be selected in consideration of cosmetic aspects, prognosis, functional prognosis, resectability, and possibility of recurrence and complications.”, despite limited scientific grounds. If complete resection of the lesion is possible, surgical resection may be performed as the first-line treatment, but the possibility of other treatments including sclerotherapy, in particular, should be evaluated according to the diverse conditions of individual patients, and surgical resection should be performed when other treatments are ineffective or when surgical resection is considered clearly superior.


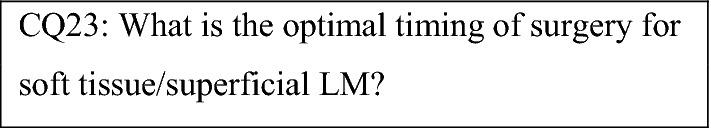


Recommendation:

It is impossible to recommend optimal timing of surgery, and judgments according to the condition of each case are necessary.

Strength of recommendation: 2 (weak)
Evidence: D (very weak)

#### Comments

[Process of preparation of recommendation]

Soft tissue/superficial LM are not malignant lesions. Emergent treatment may be necessitated by life-threatening symptoms, such as airway obstruction, but the initiation of treatment immediately after the diagnosis is generally considered unnecessary. The natural course of the disease differs considerably among individuals, particularly, in infancy, and the lesions may show a tendency of spontaneous regression but may also cause various functional problems due to rapid enlargement. Moreover, there are cosmetic problems characteristic of this disease in addition to functional problems, and early therapeutic effects are necessary to make social life comfortable. For these reasons, the selection of optimal timing of treatment, surgery in particular, is a major issue.

For the selection of the timing of surgical resection, conditions to obtain the best results as well as indications for resection must be evaluated, and sufficient consideration of the balance between merits and demerits depending on the timing of resection is necessary. Therefore, in this CQ, we attempted to summarize the presently available knowledge about “What is the optimal timing of surgery for soft tissue/superficial lesions?”.

<Literature search and screening>

As a result of literature search, 67 papers in Japanese and 231 papers in English were subjected to primary screening. Of these papers 5 in Japanese and 42 in English were subjected to secondary screening for this CQ. They included none with a high level of evidence, such as a systematic review and RCT, and all papers were case series or case reports. Therefore, the results and discussion in each case series were integrated in the evaluation of this CQ.

<Review of observational studies (case series)>

Defining “the optimal timing of surgery” mentioned in the CQ as “the timing of surgery at which good results can be obtained”, we aimed to evaluate the timing of surgery at which resection is effective, problems, such as complications are few, and, i.e., “the best results” can be obtained as a whole. Conditions must be evaluated on the basis of the timing in addition to the effectiveness of surgery, but objective judgments were considered difficult in this evaluation. However, as it was considered possible to obtain information about the age and time of surgery from the literature reviewed in the previous CQs concerning the effectiveness, papers that evaluated the age at surgery were searched.

### Results of review

Despite a careful review of the literature by secondary screening, there was no paper that analyzed cases from the viewpoint of optimal timing of surgery. There was information concerning the age at surgery, but its appropriateness was not evaluated. Papers that mentioned the timing of surgery are shown below.

Concerning the timing (age) of surgery, unless the size of the lesion is small or there are symptoms that require urgent treatment, such as respiratory disturbance, it is recommended to delay surgery until the age of 3 years by expecting spontaneous regression or for the ease of identification of surrounding structures during surgery, ease of control of bleeding, and less problems with postoperative management [294]. There was also a paper [295] that suggested the necessity of the determination of the time of surgery in consideration of problems that change with age including the priority of securing the airway and appropriate nutritional management in neonates with head and neck and giant lesions, control of hemorrhage and infection and measures to prevent dysarthria and dental problems in infants, and skeletal and cosmetic problems in school-age children, although it did not mention the optimal timing of surgery.

However, there was no paper that positively recommended resection without considering the time after the diagnosis or grounds for such a recommendation.

<Summary>

As a result of literature search for evaluating the CQ, “What is the optimal timing of surgery for soft tissue/superficial LM”, there were papers that mentioned the timing of surgery, but none of them objectively evaluated its appropriateness. Therefore, no suggestion about the appropriate timing of surgery was obtained from the literature available at present, but there were a few papers suggesting that the decision to perform surgery should be made carefully.

Similar to the previous CQ, soft tissue/superficial LM of which the background vary in individual cases, and it is difficult to uniformly evaluate the effectiveness of resection. In clinical practice, in addition to medical reasons, social reasons including school attendance are considered to largely influence the decision of the time of resection. The results of RCTs are necessary to obtain objective data, but it is practically very difficult to arrange an RCT fulfilling the above conditions.

While this CQ is a very important issue for patients and families as well as clinicians, there has not been objective evaluation of the optimal timing of surgery in the past. Presently, as quick decisions to perform surgery should be avoided, this guideline proposes, “The optimal timing of surgery cannot be decided in general, and judgments according to the condition of each case are necessary.”


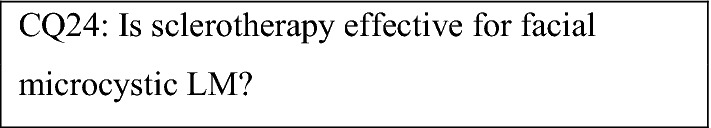


Recommendation:

A wide range of drugs are used for sclerotherapy. Although comparison among drugs has not been made, and consensus regarding the methods or frequency of their administration has not been reached, improvements are observed after sclerotherapy in various symptomatic, functional, and cosmetic (esthetic) aspects. However, complications including functional impairment have also been reported.

Strength of recommendation: 2 (weak)
Evidence: D (very weak)

#### Comments

[Process of preparation of recommendation]

[Literature search and screening]

Concerning this CQ, 35 papers in Japanese and 92 papers in English (60 from PubMed, 32 from Cochrane) were retrieved. After their primary screening, 6 in Japanese and 18 in English were subjected to secondary screening concerning this CQ. Although they included 3 RCTs, many of the other papers were case series or case reports. Therefore, in the evaluation of the draft recommendation concerning this CQ, the results and discussion in each RCT and case series were integrated. While the evidence is scarce, the papers judged to be useful for the preparation of the draft recommendation are presented as review data.

[Review of case series]

As a result of literature screening, it was found that the effectiveness of sclerotherapy for facial microcystic LM has been evaluated from the following viewpoints.

1. Treatment responses
  A. Size
  B. Symptoms
  C. Functions
  D. Cosmetics
2. Complications

The contents of the accounts concerning the effectiveness of sclerotherapy are summarized according to these viewpoints.

However, there were few reports that exclusively analyzed facial (and microcystic) LM, and lesions of the neck and other regions as well as the face were evaluated or different types of LM, such as cystic and mixed types, were reported together. In addition, the definition of the cavernous type and standard procedure of sclerotherapy (method and number of administrations) varied among reports, and these differences in the background should be considered in the evaluation of the effectiveness of sclerotherapy.

The sclerosing agents used for the literature search ranged widely from OK-432, bleomycin, ethanol, doxycycline, and sodium tetradecyl sulfate (STS). However, as none of the papers reviewed for the preparation of these guidelines evaluated differences in the effectiveness for facial microcystic lesions among drugs or the method or number of administrations of each drug, these evaluation items were excluded in discussing this CQ.

1. Responses
2. Size

Many of the papers that referred to the regression rate of the lesion classified the responses into (1) excellent or complete (regression rate ≥ 90%), (2) good or substantial (regression rate ≥ 50% and < 90%), (3) fair or intermediate (regression rate ≥ 20% and < 50%), and (4) poor or none (regression rate < 20%).

Although there was no paper that collected cases of facial lesions alone, Yang et al. [299] reported that the regression rate after sclerotherapy was ≥ 90% in 19 (63%) of the 30 patients with head and neck lesions and ≥ 50% in 10 (33%). In addition, the regression rate was reported to be ≥ 50% in 18 (85.7%) of the 21 patients with head and neck lesions by Alomari et al. [300] and in 30 of the 31 patients to be ≥ 50%, who included those with mixed type lesions, by Chaudry et al. [301]

Smith et al. [302] reported that none showed a response (complete or substantial) in 17 patients who underwent sclerotherapy, some of whom had mediastinal lesions. Giguere et al. [303] also reported that all 5 patients with head and neck lesions showed no response (poor) to the therapy. While these studies were RCTs evaluating the time of sclerotherapy, the results suggest that sclerotherapy is not effective for microcystic lesions regardless of the time of treatment.

There was no paper that compared sclerotherapy and resection for facial microcystic LM.

B. Symptoms

There is no literature that evaluated this item based on objective data, and few reports referred to symptoms themselves. The information was limited to the report by Chaudry et al. [301] that symptoms disappeared after sclerotherapy using bleomycin in 75% of the patients who complained of pain and a few case reports [304, 305] that symptoms, such as hemorrhage and respiratory impairment, were relieved after sclerotherapy.

C. Functions

Ravindranathan et al. [306] performed sclerotherapy in 3 patients with diffuse microcystic lesions extending from the face to the tongue and pharynx and reported that respiratory impairment and swallowing disorder due to airway obstruction observed before treatment were mitigated. Poonyathalang et al. [307] administered STS to a patient with orbital lesions primarily complaining of visual defect and reduced visual acuity due to retrobulbar hemorrhage and reported alleviation of the symptoms, but relevant literature was scarce similar to that concerning symptoms.

D. Cosmetic aspects

Cosmetic improvements are difficult to evaluate objectively. Poonyathalang et al. [307] administered STS to 3 patients with orbital lesions with exophthalmos as the primary symptom and reported improvement by measuring the degree of protrusion before and after the treatment. There have also been reports of objective assessment based on the degree of satisfaction in the patients’ families. According to Chaudry et al. [301], all patients with head and neck lesions (9 with microcystic lesions, 22 with mixed lesions) and their families reported improvements in the size and appearance of the lesions. In addition, Alomari et al. [300] treated 32 patients with mostly microcystic but including some cystic LM of the head and neck region by sclerotherapy and reported improvements compared with the condition before treatment by the families of 26 patients (81.3%).

2. Complications

As complications in the facial region, there are a large number of reports [299, 301, 307–312] of transient complications associated with sclerotherapy, such as fever, local swelling and pain, intracystic hemorrhage, and infection, although the lesions were poorly characterized in some reports. In addition, complications considered to have been caused by the effect of treatment, such as ulcer of the oral mucosa and tongue, facial nerve paralysis, leakage of saliva, and respiratory insufficiency due to airway obstruction, have been occasionally reported [303, 306, 307]. There have also been reports [307, 313, 314] of an elevation of the intraorbital pressure, exophthalmos, intraorbital hemorrhage, corneal damage, and external ocular muscle paralysis due to enlargement of the mass after sclerotherapy for ocular LM. There was also no literature showing the incidence of complications in facial microcystic LM.

As complications caused by sclerosing agents, skin ulcer and necrosis and nerve damage due to ethanol leakage, hypotension during anhydrous ethanol injection, and epidermal detachment due to doxycycline have been reported [300, 315]. However, there was no report of serious complications due to OK-432. Pulmonary fibrosis is widely known to be a complication of bleomycin, but, according to Chaudry et al. [301] and Yang et al. [299], impairment of respiratory function does not occur at a dose routinely employed for sclerotherapy.

[Summary]

In evaluating the CQ, “Is sclerotherapy effective for facial microcystic LM?”, analysis was performed from the viewpoints of responses to the treatment in terms of symptoms, functions, and cosmetic (esthetic) aspects and complications, but few papers with a high level of evidence were found. While the degree of regression of the lesions by sclerotherapy varied widely, the size-reducing effect of the therapy was consistently small unlike that in cystic lesions. Some papers referred to symptoms, functional outcome, and cosmetic improvement, but they were insufficient for general discussion of sclerotherapy for facial microcystic LM. As complications characteristic of sclerotherapy, serious impairment may be caused by leakage of the sclerosing agent (ethanol, in particular), and this point needs attention. Based on the above observations, it is difficult at present to evaluate indications for sclerotherapy against microcystic LM by formulating criteria. Therefore, for the future, it is considered necessary to evaluate the usefulness of sclerotherapy addressed by this CQ by designs such as RCT.


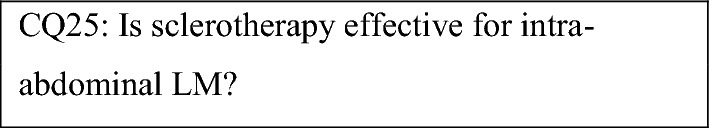


Recommendation:

Although there are many reports that sclerotherapy is useful, there is the risk of complications, and careful judgments about matters including the resectability of the lesion and selection of the sclerosing agent are necessary.

Strength of recommendation: 2 (weak)
Evidence: D (very weak)

#### Comments

[Process of preparation of recommendation]

LM is the most frequent lymphatic vessel disorder of the abdomen. Intra-abdominal lesions are estimated to account for 10–20% of all LM, and the selection of treatment is difficult depending on the site of the lesion. While surgical resection is expected to be effective, less invasive treatments are considered necessary in view of stress to the patient and the possibility of severe complications such as lymphatic fluid leakage and bowel obstruction. Sclerotherapy, which is a major treatment for LM, is considered to be less invasive than surgery. Although positive therapeutic effects are expected, sclerotherapy is known to induce marked inflammation. Whether it can be performed safely without negative effects including complications and its long-term effects are major clinical concerns. In addition, what therapeutic effects are expected or what complications should be anticipated after sclerotherapy for the intra-abdominal lesion is also unclear. Therefore, the CQ, “Is sclerotherapy effective for intra-abdominal LM?”, was formulated, and knowledge available at present was compiled.

<Literature search and screening>

As a result of literature search, 19 papers in Japanese and 38 papers in English (32 from PubMed, 6 from Cochrane) were subjected to primary screening. Of these papers, 2 in Japanese and 9 in English were subjected to secondary screening concerning this CQ. They included no papers with a high level of evidence, such as systematic reviews and RCTs, and all were case series or case reports. Consequently, the results and discussion in each case series were integrated in the evaluation of this CQ.

<Review of observational studies (case series)>

The literature concerning the effectiveness of sclerotherapy for intra-abdominal LM was reviewed from the viewpoints of (1) therapeutic effects (decrease in lesion size, symptoms) and (2) complications.

The drugs used for sclerotherapy ranged widely from OK-432 to bleomycin, ethanol, doxycycline, STS, acetic acid, steroid/tetracycline, and 50% glucose solution. According to our review, there was no paper that evaluated the differences in effectiveness of sclerotherapy in the abdomen according to the drug type or administration method or number of administrations of each drug.

### Results of review

1. Therapeutic effects
2. Regression rate of the lesion

Regression of lesions of intra-abdominal LM by sclerotherapy was mentioned in 5 papers [288, 316–319]. According to the report by Chaudry et al. [316], the reduction rate was ≥ 90% in 7 and ≥ 20% in 1 of the 10 patients with LM of the mesentery and retroperitoneum treated with doxycycline, and evaluation using imaging examination was not performed in 2 cases. The patient who showed a low regression rate had a mixed type of cystic and cavernous lymphangiomas, and the other patients had cystic lesions. Oliveira et al. [317] reported that the lesion regressed by 70% in 1 of the 2 patients with cystic lymphangiomas treated with OK-432. Won et al. [318] reported 1 patient who showed complete disappearance of cystic retroperitoneal lesions after sclerotherapy using acetic acid. Shiels et al. [319] reported that cystic lesions responded to sclerotherapy using STS and ethanol in 2 patients, but there was no mention about the reduction rate. However, according to Alqahtani et al. [288], no effect was observed in 10 patients who underwent sclerotherapy using steroid/tetracycline or 50% glucose solution.

B. Symptoms

There were 3 papers that referred to symptoms of patients treated by sclerotherapy for intra-abdominal LM [316, 317].

According to Chaudry et al. [316], of the 10 patients who underwent sclerotherapy, 3 had chronic abdominal pain, 3 had acute abdominal pain, 1 had fever/chill, 1 had anemia, and 2 had palpable masses, but the symptoms were alleviated by treatment in all patients, and no recurrence was noted.

Oliveira et al. [317] reported that sclerotherapy was performed in a patient with a palpable mass and in 1 with a palpable mass, abdominal compartment syndrome, and a poor general condition. While the condition was alleviated in the patient who only showed a palpable mass after 2 courses of OK-432 sclerotherapy, but the treatment was changed to surgery in the patient who had abdominal compartment syndrome because of enlargement of the mass due to intracystic hemorrhage.

2. Complications

Three papers specifically mentioned complications of sclerotherapy for intra-abdominal LM. There was no report of deaths due to treatment-related complications. Oliveira et al. [317] treated 3 patients by sclerotherapy using OK-432 and reported that one of them developed subbowel obstruction after the treatment and another required emergency surgery due to exacerbation of abdominal compartment syndrome induced by intracystic hemorrhage. Chaudry et al. [316] reported that doxycycline used for sclerotherapy leaked into the retroperitoneal space in 1 of the 10 patients but that the lesion regressed without any particular problem. Won et al. [318] performed sclerotherapy using acetic acid in 1 patient with retroperitoneal cystic lymphangioma. Although pain and hematuria were observed, they concluded that the relationship of hematuria with the therapy was unclear, because it was observed during menstruation.

### Limitations

Sclerotherapy was often performed before, after, or during surgical resection, and papers that reported the results of sclerotherapy alone were few. There was no paper that directly compared observation without treatment, sclerotherapy, and surgical resection. Few papers analyzed intra-abdominal lesions alone, and many papers included lesions in other areas or evaluated lesions in different intra-abdominal regions including the mesentery, retroperitoneum, and viscera collectively.

Moreover, differences in properties of LM, such as cystic, cavernous, and mixed types, their definitions, criteria for the selection of sclerotherapy (combination with surgery, types of sclerosing agents and methods of their use, number of administrations) varied among papers, and few papers evaluated these matters separately.

Such differences in the patient background and contents of treatment must be considered in evaluating the effectiveness of sclerotherapy. In evaluating this CQ, particularly, differences in morphology of LM and sclerosing agents were excluded.

<Summary>

The CQ, “Is sclerotherapy effective for intra-abdominal LM?” was evaluated from the viewpoints of therapeutic effect, symptoms/functions, and complications, but no paper with a high level of evidence was found. While sufficient regression of the lesion and alleviation of symptoms were achieved by sclerotherapy in some patients, the response rate varied among reports, and information was insufficient for general discussion of sclerotherapy. Concerning treatment-related complications, there have been reports of bowel obstruction associated with sclerotherapy, and attention to this condition as well as intracystic hemorrhage is considered necessary. However, there was no report of chylorrhea, which was reportedly caused by surgery.

Based on the above observations, it is presently difficult to determine indications for sclerotherapy in intra-abdominal LM by setting up criteria, but as there was no literature that strongly ruled out intra-abdominal LM as indications of sclerotherapy, these guidelines propose, “Although there are many reports that sclerotherapy is useful, there is the risk of complications, and careful judgments about matters including the resectability of the lesion and selection of the sclerosing agent are necessary.” For the future evaluation of this CQ, validation by a design with a high level of evidence, such as RCT, is considered necessary.


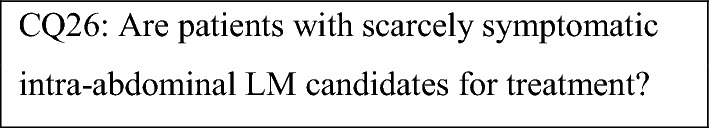


Recommendation:

Since there is risk of treatment-related complications, it is proposed to consider therapeutic intervention when the lesion tends to enlarge or has become symptomatic.

Strength of recommendation: 2 (weak)
Evidence: D (very weak)

#### Comments

[Process of preparation of recommendation]

Intra-abdominal LM occasionally presents with severe symptoms, such as abdominal pain, giant mass, and bowel obstruction, but may also be asymptomatic and detected incidentally. Lesions may gradually enlarge and cause serious symptoms due to infection and intraluminal hemorrhage.

Under such circumstances, whether patients with nearly asymptomatic intra-abdominal LM should be aggressively treated, or when intervention should be optimally performed during their long follow-up period are major problems that pose clinical dilemma. Therefore, the CQ, “Are patients with scarcely symptomatic intra-abdominal LM candidates for treatment?”, was formulated, and knowledge available at present was summarized.

<Literature search and screening>

As a result of literature search, 206 papers in Japanese and 237 papers in English (230 from PubMed, 7 from Cochrane) were subjected to primary screening. Of these papers, 6 in Japanese and 9 in English were subjected to secondary screening concerning CQ 26. They included no papers with a high level of evidence, such as a systematic review or RCT, and many of them were case series or case reports. Since 7 papers among them described asymptomatic LM, their results and discussions were integrated to answer the CQ.

<Review of observational studies (case series)>

Seven papers [316, 317, 320–324] among reviewed literature described asymptomatic LM. Fifteen cases reported in these papers were considered to have actually presented few symptom (including asymptomatic patients who were incidentally detected by imaging studies to have intra-abdominal masses at the sites as greater omentum, mesentery and retroperitoneum).

The literature was screened, and papers addressing issues concerning therapeutic intervention for scarcely symptomatic intra-abdominal LM including “What symptoms they may present with if they are left untreated?”, “By what studies and how often should they be examined?”, and “What other treatments are available and how serious are complications or risk of each treatment?” were reviewed.

### Results of review

From the literature reviewed, symptoms of intra-abdominal LM (abdominal pain, bowel obstruction, torsion, infection, hemorrhage, vomiting/sucking difficulty, frequent urination and abdominal mass [320–326]) are considered to be dependent on factors such as site, size and age. It is necessary to determine risk factors by stratification of these factors in the future [320, 322, 325].

Reported complications in treated cases include recurrence that required re-treatment [321], bowel obstruction [317, 323, 324], chylous ascites [324, 326], embolism [317], hemorrhage [317] and wound infection. Embolism of the inferior vena cava after surgery [317] and abdominal compartment syndrome after adhesion therapy [317] were reported as severe complications. It deserves special attention that, if surgical resection is performed for mesenteric LM, the intestine may have to be resected with the lesion [326].

While there have been reports [320, 322] that intra-abdominal LM with few clinical symptoms regressed during follow-up, they may become symptomatic later (as observed in many case reports). For that reason, the opinion that intervention should not been chosen during the follow-up until the lesion enlarges or new symptoms appear was frequently described.

### Limitations

It should be noted that many asymptomatic cases may be left unreported and some asymptomatic lesions that had been detected were treated. There is no study with a high level of evidence indicating explicit criteria concerning the age, site or situation about whether intervention should be made for asymptomatic intra-abdominal LM.

<Summary>

The necessity of treatment of a patient with intra-abdominal LM with few symptoms should be determined after evaluating the balance between the risk of treatment and non-treatment considering its site and size as well as patient age. However, since research on indications for treatments has been insufficient so far and serious complications after treatment have been reported, deliberate evaluation for each patient is mandatory. When observation is selected, periodic imaging studies are recommended to optimize therapeutic intervention by detecting enlargement of the lesion. Also if any symptom has developed during follow-up, intervention should be considered. For these reasons, the recommendation, “Since there is risk of treatment-related complications, it is proposed to consider therapeutic intervention when the lesion tends to enlarge or has become symptomatic.” was adopted.


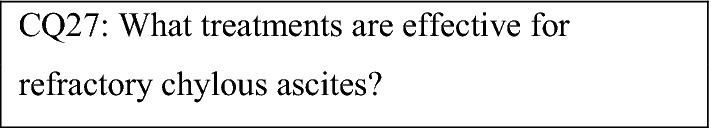


Recommendation:

Conservative treatments, such as fasting, high-calorie infusion, and medium chain triglyceride (MCT), should be performed first, but, if they are ineffective, drug treatment, sclerotherapy, and surgery may also be considered.

Strength of recommendation: 2 (weak)
Evidence: D (very weak)

#### Comments

[Process of preparation of recommendation]

Refractory chylous ascites causes loss of large amounts of protein and lymphocytes, decreases in the blood lipid levels, and abdominal pain, unpleasantness, and dyspnea due to abdominal distention and markedly reduces the quality of life. The cause of ascites often remains unknown. Treatment of chylous ascites may require drainage to avoid abdominal distention. It is a very important point for clinicians to make proper judgments by understanding treatments and their effects and demerits. Therefore, it is considered beneficial to collect information about chylous ascites over a long period and compile guidelines. For this purpose, the presently available knowledge was collected by formulating the CQ, “What treatments are effective for refractory chylous ascites?”

<Literature search and screening>

As a result of search, 161 papers in Japanese and 728 papers in English (564 from PubMed, 164 from Cochrane) were subjected to primary screening. Of these papers, 15 in Japanese and 12 in English were subjected to secondary screening for CQ 27. They included none with a high level of evidence, such as systematic reviews and RCTs, and consisted of 1 multicenter and 2 single-center case series and case reports. Consequently, we used the results and discussion of 27 papers judged for the preparation of the draft recommendation were integrated although evidence was insufficient for the evaluation of this CQ.

<Review of observational studies (case series)>

As for causes of chylous ascites, congenital chylous ascites [327–342], idiopathic chylous ascites [328], chylous ascites after laparotomy [343–346], protein-losing enteropathy [345], LM [347, 348], lymphangiectasis [349, 350], lymphangiomatosis [351, 352], and lymphatic dysplasia [353] were reported. None of the papers evaluated treatments according to the cause.

When treatments are categorized, conservative treatments (fasting, high-calorie infusion, MCT), drug treatments, sclerotherapy, and surgical treatment were performed.

### Results of review

The results of review are presented below according to the treatment.

1. Conservative treatments

Whether the amount of ascites changes by fasting should be checked first.

High-calorie infusion is often used with fasting, and since there was no report that ascites increased under the effect of high-calorie infusion according to our review, it is recommended for nutritional support during fasting. In the multicenter case series reported by Bellini et al. [327], high-calorie infusion/total parenteral nutrition was performed in 15 patients without adverse effects.

Medium chain triglyceride was used before, after, and during treatment [327, 328, 330–335, 337, 339–341, 343, 345, 346, 348–352]. In the multicenter case series by Bellini et al. [327], MCT was reportedly performed in 14 patients without adverse effects.

2. Drug treatments

In drug therapy for chylous ascites, primarily octreotide (a long-acting somatostatin analogue) was used, and no report that discussed the effectiveness of other drug therapies was found by the present literature search.

In the multicenter case series by Bellini et al. [327], octreotide was administered to 6 of the 16 patients with chylous ascites for 8–38 days, and a decrease in chylous ascites was reported in all of them. In the single-center case series by Huang et al. [344], 2 of the 4 patients with chylous ascites treated by high-calorie infusion and octreotide administration were reported to have shown a decrease in ascites within 10 days. However, there has been a report [330] that no effect was observed despite the administration of octreotide for 3 weeks. Concerning the dose of octreotide, it was administered at 1 μg/kg/h [327], at 3 μg/kg/h [332], began to be administered at 0.5 μg/kg/h and increased to 10 μg/kg/h by 1 μg/kg/h [329], administered by continuous intravenous infusion at 0.5–2.0 μg/kg/h [333], and began to be administered by subcutaneous injection at 2.5 μg/kg 2 times/day and increased every 2 days to 8 μg/kg 2 times/day [330]. Regarding the time of the beginning of administration, the administration was started as no improvement was observed in chylous ascites after conservative treatments for 2 weeks [330, 334], and as chylous ascites was alleviated by conservative treatments but was exacerbated again [333]. No adverse effects of octreotide administration were noted in the present review of the literature. Thus, no control study that evaluated the effect of octreotide on chylous ascites was found by the present literature search, and the level of evidence concerning the efficacy is low, but as there are case series and many case reports that chylous ascites was reduced by octreotide administration, it appears reasonable to consider drug treatment using octreotide for chylous ascites that does not respond to conservative treatments.

3. Sclerotherapy

Sclerotherapy was performed in 6 patients in 5 case reports [339, 347, 349, 351, 352]. The sclerosing agent was OK-432 in 5 of the 6 patients and was Beta-Isodona-solution^®^ (MUNDIPHARMA GmbH, Cambridge, UK) in 1 [349]. OK-432 was locally injected into the lesion in 4 [347, 351, 352], administered intraperitoneally in 1 [352], and administered via the drain in 2 [347, 352]. Concerning sclerotherapy, the number of reported cases that were reviewed was limited, and further accumulation of cases is considered necessary to establish its usefulness.

4. Abdominal drainage, abdominal puncture, and surgical treatment

Abdominal drainage and abdominal puncture are performed when organ compression symptoms (compartment syndrome and respiratory insufficiency) due to abdominal distention are present or possible or when the drain is inserted postoperatively. However, drainage itself cannot improve chylous ascites, and treatments, such as infusion, blood preparations, and blood transfusion, are necessary to supplement the ascites lost due to drainage [327, 330–333, 337–340, 343, 345–347, 349, 351, 352].

Surgical treatment is reported to be frequently performed after conservative or drug treatments. According to the single-center case series by Zeidan et al. [343], surgical treatment was performed in patients who responded poorly to conservative treatments for over a mean of 25.3 days. In other reports, surgical treatment was performed after conservative treatments for 1–3 months [328, 329] and in patients with congenital chylous ascites 1–4 months after birth [330, 334, 350]. Since it is often impossible to identify the leakage site of chylous ascites [330], attempts to identify the leakage site by orally administering a lipophilic dye (Sudan black, Sudan III) before operation. [328, 329, 336, 343] When the leakage site can be identified, ligation, suturing, clipping, and cauterization have been performed [328, 334, 336, 343, 350]. In addition to reports of the usefulness of techniques to stop leakage, such as applying or sprinkling fibrin glue at the leakage site of chylous ascites or over the surrounding retroperitoneum [329, 331, 343, 350] and applying a patch of oxidized cellulose/resorbable local hemostatic agent [331, 343], there have also been reports of peritoneovenous shunting [349, 353] and peritoneoamniotic shunting for fetal cases [338].

There was no large clinical study in the past literature. Therefore, although the level of evidence is low, we consider that surgical treatment is recommendable for chylous ascites that does not respond to conservative or drug treatments, because it has been performed in case series and case reports for chylous ascites that did not respond to conservative or drug treatments for over about 1 month. Although techniques to enhance the response rate of surgical treatment, such as identifying the leakage site by using a lipophilic dye and applying fibrin glue or a patch of oxidized cellulose/resorbable local hemostatic agent, have been attempted, there are only case series and case reports, and none of the papers retrieved by the present literature search evaluated their usefulness.

### Limitations

There was no literature that defined refractory chylous ascites based on the duration of illness or treatment responses. Therefore, we extracted and summarized factors that were considered to contribute to clinical refractoriness, such as the duration of illness and treatment responses, in each paper related to the treatment for chylous ascites. Also, as the cause of chylous ascites varies widely, the therapeutic effect is expected to differ depending on the cause, but no paper that was reviewed evaluated treatments according to the cause. Therefore, in the present evaluation, the statements are limited to treatments and their effects regardless of the cause.

<Summary>

It was difficult to comprehensively discuss treatments, because its cause varied widely, and treatments for various causes were performed. Therefore, treatments were classified into conservative treatments (fasting, high-calorie infusion, MCT), drug treatments (octreotide), sclerotherapy, abdominal drainage, abdominal puncture, and surgical treatment, and the effects of each treatment were evaluated.

Treatments effective for refractory chylous ascites can be summarized as follows with the understanding that they may depend on the cause and that the level of evidence of the available reports concerning treatments and their effects is low. Conservative treatments, such as fasting, high-calorie infusion, and MCT, should be performed first because of the rareness of adverse effects. In patients who respond insufficiently to conservative treatments, drug treatments using octreotide can be considered as there have been case series and many case reports. Concerning sclerotherapy, the number of reported cases is small, and further large clinical studies will be needed to confirm its usefulness. Abdominal paracentesis and surgical treatments may be considered for chylous ascites that does not response to conservative or drug treatments for about 1 month.

Thus, the draft recommendation is “Conservative treatments, such as fasting, high-calorie infusion, and MCT, should be performed first, and, if they are ineffective, drug treatments, sclerotherapy, and surgical treatments may be considered”. However, evaluation of this CQ by a design with a higher level of evidence, such as RCT, is considered necessary for the future.


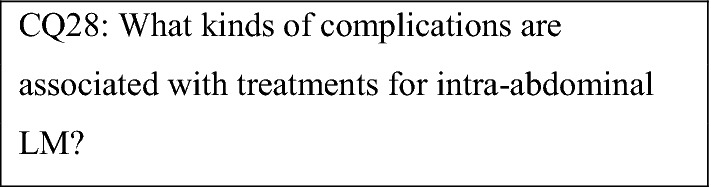


Recommendation:

Complications associated with sclerotherapy for intra-abdominal LM include bowel obstruction, hemorrhage, pain, hematuria and chylous ascites. Surgical treatment of the disease can be associated with serious complications such as occlusion of the inferior vena cava and massive resection of the intestine as well as more common, wound infection, bowel obstruction, hemorrhage and chylous ascites.

Strength of recommendation: No recommendation
Evidence: D (very weak)

#### Comments

[Process of preparation of recommendation]

Patients with intra-abdominal LM are treated with various modalities from non-surgical therapy to surgical procedures. Treatment modality is selected depending on the patient’s state. Therefore, it is necessary for the clinician, patient, and family to share information concerning complications that may be associated with treatments for implementing them smoothly. However, there are no resources that give a clear answer to this problem, and both clinicians and patients may become disconcerted. Therefore, the CQ “What kinds of complications are associated with treatments for intra-abdominal LM?” was formulated, and information available at present was accumulated and integrated for the answer.

<Literature search and screening>

As a result of literature search, 203 papers in Japanese and 602 papers in English (593 from PubMed, 9 from Cochrane) were subjected to primary screening. Of these papers, 23 in Japanese and 27 in English were subjected to secondary screening concerning this CQ. They included no papers with a high level of evidence, such as systematic reviews or RCTs, and all of them were case series or case reports. To answer CQ 28, the results and discussion in each case series were integrated.

<Review of observational studies (case series)>

Complications in the CQ were evaluated by defining them as those encountered when patients with intra-abdominal LM were treated, and reports on sclerotherapy and surgery were reviewed.

### Results of review

1. Complications associated with sclerotherapy

Sclerotherapy using OK-432 was reported to be associated with bowel obstruction and hemorrhage for mesenteric LM [317], and chylous ascites for retroperitoneal LM [326]. Sclerotherapy using acetic acid was reported to be associated with pain and hematuria in patients with retroperitoneal LM [318].

2. Complications associated with surgical procedures

Complete resection of both mesenteric and retroperitoneal LM by laparotomy was reported to be associated with wound infection [324, 354] and bowel obstruction [323, 354, 355] as common complications. There were reports of serious complications such as occlusion of the inferior vena cava [317] and massive resection of the intestine was necessary due to diffuse infiltration of the LM tissue to the intestinal wall [356].

In a report [357] about complications associated with complete laparoscopic resection of intra-abdominal LM by Tran et al., resection was attempted in 47 patients, and conversion to laparotomy was necessary in 3 (6.4%) due to tight adhesion in 2 and intraoperative hemorrhage in 1.

Partial resection by laparotomy was reported to be associated with persistent ascites over a long period that was refractory to the treatment [355].

### Limitations

Patients with intra-abdominal LM are treated with various modalities including sclerotherapy and surgical procedures. Modalities were combined in many cases, and complications are often reported as those of entire treatment without more detail information about those associated with individual treatment.

<Summary>

For answering the CQ, “What kinds of complications are associated with treatments for intra-abdominal LM?”, no literature with a high level of evidence was found, but foreseeable complications were listed from many case reports. Bowel obstruction, hemorrhage, pain, hematuria, and chylous ascites were reported as complications of sclerotherapy. Serious conditions, such as occlusion of the inferior vena cava and massive resection of the intestine, as well as common complications, such as wound infection, bowel obstruction, hemorrhage and chylous ascites were reported as complications after surgical procedures.

Although the incidences and differences in complications in respect of the site and histological type are not shown in the literature, each patient with intra-abdominal LM should be treated with sufficient evaluation of the site, size and symptoms. In addition, treatment must be implemented with sufficient understanding of the possible complications.

Thus, we propose “Complications associated with sclerotherapy for intra-abdominal LM include bowel obstruction, hemorrhage, pain, hematuria, and chylous ascites. Surgical treatment of the disease can be associated with serious complications such as occlusion of the inferior vena cava and massive resection of the intestine as well as more common, wound infection, bowel obstruction, hemorrhage and chylous ascites.” as a recommendation draft.


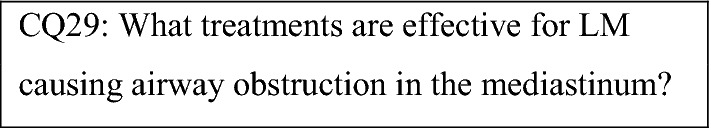


Recommendation:

Sclerotherapy is effective for macrocystic lesions, and surgical resection is effective for microcystic lesions. However, as the complication rate is relatively high, treatments should be selected according to the condition of each case.

Strength of recommendation: 2 (weak)
Evidence: D (very weak)

#### Comments

[Process of preparation of recommendation]

Among LM, those that may cause airway obstruction due to their sites are life-threatening. Lesions in the mediastinum cause respiratory disorders if they physically compress the trachea or bronchi and obstruction the airway or markedly protrude into the thoracic cavity and narrow it.

In such situations, aggressive and effective treatment is necessary, but the therapeutic approach must be selected carefully in consideration of the relationship of the lesion with the important organs around it such as the large cardiac vessels, mediastinal nerve, and thoracic duct. However, the judgment is often difficult in clinical settings.

Therefore, the CQ, “What treatments are effective for LM causing airway obstruction in the mediastinum?” was formulated, and the presently available knowledge concerning matters including the risk of complications and prognosis of treatments, such as surgical resection and sclerotherapy, was summarized.

<Literature search and screening>

As a result of literature search, 134 papers in Japanese and 227 in English (226 from PubMed, 1 from Cochrane) were subjected to primary screening. Of these papers, 5 in Japanese and 16 in English were subjected to secondary screening concerning this CQ. Since they included none with a high level of evidence, such as a systematic review or RCT, and all were case series or case reports, the results and discussion in each case series were integrated.

<Review of observational studies (case series)>

By screening of the literature, the following approaches were found for the treatment of LM in the mediastinum.

Therapeutic options are surgical resection, puncture and drainage, sclerotherapy (OK-432, bleomycin, ethiblock, anhydrous ethanol), drug treatments (Chinese herbal medicines such as *eppikajutsuto* and *ogikenchuto*), and no treatment. Of these approaches, surgical resection and sclerotherapy using OK-432 have been evaluated in a relatively large number of cases, and reports of other therapy had extremely limited number of cases, e.g., reports of only 1 case.

### Results of review

Boardman et al. [358] reported that, of the 97 patients with LM of the head and neck region, surgical treatments were necessary in 6 of the 12 patients with mediastinal lesions, that complications of surgery occurred in 4 of the 6 patients, and that long-term nerve damage was observed in 3 of them. In addition, they reported that management by tracheotomy was necessary in 15% of all patients. Complete or nearly complete remission was observed in 92% of the patients, but they suggested that surgical treatments should be indicated only when there is airway obstruction or there is the risk of it, because surgical treatment of mediastinal lesions frequently causes complications.

Park et al. [359] reported that they surgically resected mediastinal LM in 12 patients. Seven of them had dyspnea, and 3 were asymptomatic, but they were all judged to have indications for surgery due to symptoms or the tendency of the lesions to enlarge. A total of 5 recurrences were observed in 4 patients (33%) during a mean period of 3.6 years after the initial surgery, and all underwent by re-resection. No perioperative death was observed, and, in a total of 25 cases including past cases, the overall survival was not different compared with that in healthy individuals over a follow-up period of 11.5 years.

Smith et al. [302] performed local injection of OK-432 in 16 patients with mediastinal LM and reported ≥ 60% regression of the lesion in 13 (81%). They also mentioned treatment responses according to the histological types and, by reporting responses (complete or nearly complete remission) in 94% of those with macrocystic lesions, 63% of those with mixed lesions, but 0% in those with microcystic lesions, suggested a macrocystic lesion to be a good indication for sclerotherapy using OK-432. Although not from the viewpoint of airway obstruction, they reported that treatment using OK-432 was more effective than surgical resection and less frequently caused serious complications.

### Limitations

There have been no papers that directly analyzed treatments effective for mediastinal lesions expected to cause airway obstruction, and many papers reported cases of mediastinal lesions that responded to treatments. Therefore, we simply extracted matters relevant to this CQ from these reports.

<Summary>

There was no literature with a high level of evidence concerning effective treatments for LM in the mediastinum causing airway obstruction. A few case reports that referred to the effects of surgery and sclerotherapy were observed, but it was difficult to present objective and specific figures concerning their effectiveness or safety. However, according to the available information, it should be noted that favorable responses have been obtained by OK-432 local injection in macrocystic lesions and that complications due to surgical resection are likely to occur relatively frequently.

From these observations, we consider the following to be a therapeutic approach that can be proposed: “Sclerotherapy, such as that by local injection of OK-432, should be considered for macrocystic lesions, and, for lesions that are technically difficult to treat by sclerotherapy or microcystic lesions, surgical resection should be considered with attention to complications. In addition, it is necessary to pay attention to the appearance of respiratory disturbances before and after these treatments and to constantly evaluate indications for securing the airway by intratracheal intubation or tracheostomy.” Therefore, at present, we recommend, “Sclerotherapy is effective for macrocystic lesions, and surgical resection is effective for microcystic lesions. However, as the complication rate is relatively high, treatments should be selected according to the condition of each case.”


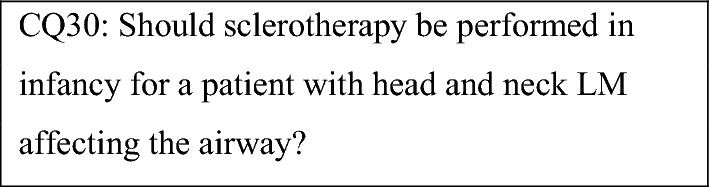


Recommendation:

In a patient with LM around the airway, there is risk of respiratory distress in infancy, while airway obstruction is likely to be exacerbated by sclerotherapy. Particularly, when risk of airway obstruction is judged to be high or when the patient has already presented symptoms, it is proposed to perform sclerotherapy with sufficient preparations including airway management.

Strength of recommendation: 2 (weak)
Evidence: D (very weak)

#### Comments

[Process of preparing recommendation]

LM of the neck, which are located in an exposed part of the body, may cause cosmetic problems that are important, but airway obstruction can particularly be a serious problem in some cases.

Sclerotherapy, which is one of the major treatment modalities, is most effective in patients with cystic LM, but swelling of the treated portion after the therapy may cause or exacerbate airway obstruction symptoms especially in neonates. The upper airway will become less vulnerable to obstruction because it becomes less frail and wider as patients grow and respiratory distress tends to be unlikely. Therefore, it is occasionally difficult to determine how a patient who does not present any obstructive symptom should be treated in infancy.

Thus, we evaluated this problem by formulating the CQ, “Should sclerotherapy be performed in infancy for a patient with the neck LM affecting airway?”

<Literature search and screening>

As a result of search, 86 papers in Japanese and 135 papers in English (130 from PubMed, 5 from Cochrane) were subjected to primary screening. Of these papers, 6 in Japanese and 20 in English were subjected to secondary screening concerning this CQ. They included 1 systematic review (SR), 1 RCT, 2 prospective studies (PS) and 1 retrospective cohort study, but all the others were case series or case reports. Therefore, the results and discussion, primarily, in these SR, RCT, PS, and retrospective cohort study, but also in other case series were integrated.

<Review of observational studies>

The literature concerning the effectiveness of sclerotherapy for head and neck LM in infancy was reviewed from the viewpoints of responses (prognosis (survival rate or mortality), size, symptoms, and cosmetic improvement) and complications.

Sclerosing agents used as keywords for the present literature search varied widely and included OK-432, bleomycin, ethanol, doxycycline, STS and fibrin glue. No paper evaluated differences in effectiveness of various agents due to their methods of administrations for lesions around the neck affecting airway. Therefore, differences among agents were excluded from the evaluation of this CQ.

### Results of review

1. Responses
2. Prognosis (survival rate or mortality)

According to the SR by Adams et al. [360], the mortality was 4.7% in 277 cases with head and neck LM. Since lesions around the airway were not the only target, and since sclerotherapy was not the only treatment modality, the paper has not quite rightly answered to the CQ. However, since patients who died were all before one year of age and their causes of death are considered to have been mostly airway problems, such as airway obstruction and aspiration due to vocal cord paralysis in 8, and as at least 1 patient is judged to have died due to complications of invasive treatment, the paper is considered to indicate the risk of this disorder during infancy.

B. Size

Many of the papers that referred to the size regression evaluated it by four categories; (1) excellent or complete (≥ 90% regression), (2) good or substantial (≥ 50% and < 90% regression), (3) fair or intermediate (≥ 20% and < 50% regression), (4) and poor or none (≤ 20% regression).

Ravindranathan et al. [306] treated 5 patients (aged 4–19 months) with cervicofacial LM by sclerotherapy using OK-432 (in addition to Fibrovein^®^ (STD Pharmaceutical Products Ltd, Hereford, UK) in 2) and reported that the responses were good in 1 (20%) (cystic), partial in 1 (20%) (cavernous), and poor in 3 (60%) (2 with cavernous lesions that required tracheotomy and 1 with cystic lesions in whom the condition improved to good after surgical resection). However, they did not mention the evaluation criteria for good, partial, and poor.

According to the report of 8 cases with head and neck LM by Leung et al. [361], all patients underwent sclerotherapy, ≥ 50% regression was observed in all patients with complete regression in 2. However, the patient age varied from 2 months to 11 years, and the types of LM were not mentioned.

Ogawa et al. [362] reported 9 patients (including 5 preschoolers and toddlers, 2 school children, and 2 adults) who underwent OK-432 sclerotherapy for the neck LM and evaluated it to be markedly effective in 8, in whom the lesions mostly disappeared, and effective in 1, who showed a ≥ 50% regression. Eight patients in whom the treatment was markedly effective consisted of 1 with mixed and 7 with cystic lesions, and the one in whom the treatment was effective had a mixed type.

Cahill et al. [315] reported doxycycline sclerotherapy in 17 patients with head and neck LM (cystic in 10, mixed in 7 (3 required tracheotomy)), and its size regression was reported to be > 90% in 7 (41.2%) (cystic in 6, mixed in 1), 75–89% in 4 (23.5%) (cystic in 2, mixed in 2), 51–74% in 4 (23.5%) (cystic in 1, mixed in 3), and 25–50% in 2 (11.8%) (mixed in 2).

Nehra et al. [311] reported doxycycline sclerotherapy in 11 patients with head and neck LM (cystic in 7, mixed in 4; aged 2 days–21 months) (later combined with surgical resection in 3). The treatment results were excellent in 5 (45.5% of all patients) and satisfactory in 2 (18.2% of all patients) among 7 patients with cystic lesions but poor in all 4 patients with mixed type lesions (36.4% of all patients). Particularly, 3 of the 4 patients with mixed type lesions required tracheal intubation shortly after birth and underwent sclerotherapy while intubated, but the effects were poor in all of them. Surgical resection was added in 1 and was under consideration in another.

C. Symptoms

According to Ravindranathan et al. [306] who reported 5 patients with cervicofacial LM (aged 4–19 months) treated with sclerotherapy using OK-432 (in addition to Fibrovein^®^ (STD Pharmaceutical Products Ltd, Hereford, UK) in 2), 4 exhibited symptoms of airway obstruction before treatment. Symptoms included dysphagia in 2 and dyspnea (including croup-like symptoms) in 4 (some both). Symptoms were alleviated by sclerotherapy in 2 out of 4 (cystic 1, cavernous 1), but tracheotomy was necessary in the remaining 2 (cavernous in both) without improvement.

In the report of 8 patients with head and neck LM and 5 patients with VM (aged 2 months–11 years) by Leung et al. [361], their symptoms noted before treatment were mass or swelling [10 patients (77%)], pain after hemorrhage [2 patients (15%)], skin discoloration (blue) [1 patient (8%)], obstructive airway symptoms [6 patients (46%)], and swallowing difficulty [1 patient, (8%)]. All symptoms were alleviated by sclerotherapy (doxycycline for LM, STS foam for VM).

Arimoto et al. [363] reported a patient with cystic LM in the neck presented 3 months after birth. The patient presented with respiratory distress at the age of 10 months due to enlargement of the LM following upper respiratory infection. While left vocal cord fixation due to the mass was confirmed by ultrasonography before treatment, aspiration of the cyst and steroid administration resulted in opening of the glottic area and regression of the mass with relief of wheezing and distress. Since they underwent sclerotherapy 2 months after the disappearance of symptoms, aspiration of internal fluid and steroid administration rather than sclerotherapy were directly effective for the alleviation of symptoms.

Kitagawa et al. [364] reported a patient with giant LM of the neck which had been prenatally diagnosed and was treated under ex utero intrapartum treatment (EXIT) by tracheal intubation after aspiration of the cyst. The lesion was reported to be refractive to subsequent sclerotherapy and tracheotomy was eventually needed.

Nehra et al. [311] reported that, among 11 patients with head and neck LM (cystic type in 7 and mixed type of cystic + cavernous in 4; aged 2 days–21 months), 3 out of 4 with mixed LM presented respiratory distress soon after birth and were managed by intubation, but that all were extubated after sclerotherapy using doxycycline (1–3 times, median: 1.6 times).

D. Cosmetic improvements

No paper has reported cosmetic results in detail. Only sporadically they mentioned about surgery for redundant skin after regression of cystic lesions by sclerotherapy.

4. Complications

Complications associated with treatment for LM around the airway have been reported in many papers. They include temporary conditions caused by sclerotherapy such as fever [292, 303, 312, 362, 365–371], local swelling [303, 312, 365, 367, 368, 370, 371] pain [288, 312, 362, 367, 370–372], hemorrhage into the cyst [288, 303, 312, 368], and infection [288, 292, 303, 310, 312, 360, 365, 372]. There also reported complications as the effects of treatment for head and neck lesions such as respiratory distress due to airway obstruction [292, 303, 306, 312, 362, 365, 366], as well as nerve palsy [288, 292, 312, 360, 365].

According to a systematic review about head and neck LM by Adams et al. [360], both nerve damage due to sclerotherapy and post-therapeutic infection were reported in 1 (0.8%) out of 123 patients. Since nerve damage and infection after surgery were observed in 12 (10.2%) and 7 (5.9%) out of 118 patients, respectively, the complication rate would be lower by sclerotherapy than by surgery.

Ogawa et al. [362] reported a 1-year- and-5-month-old patient who developed airway edema after OK-432 sclerotherapy for the cystic neck LM and necessitated tracheal intubation for 3 days and cautioned against sclerotherapy for LM around the airway in young children (particularly, those less than 2 years old).

Kudo et al. [369] also reported 2 patients aged 11 months and 1 year and 11 months who were treated with OK-432 sclerotherapy intubated in advance for fear of airway obstruction due to post-therapeutic swelling. Tomemori et al. [373] also cautioned against sclerotherapy for LM in children aged less than 2 years as in the report by Ogawa et al. [362]

On the other hand, Kudo et al. [369] reported 2 cases whose neck LM having been enlarged rapidly after suffering from measles or upper respiratory tract infection (URTI). Arimoto et al. [363] also reported a patient with cystic LM of the neck 3 months after birth, who developed dyspnea due to enlargement of the lesion after URTI at 10 months and was about to be intubated.

Regarding complications due to sclerosing agents, Cahill et al. [315] reported those by doxycycline, STS and absolute ethanol. They reported delayed complications, such as Horner’s syndrome, transient left lip weakness, right facial nerve palsy, and transient left hemidiaphragm paralysis, in addition to peri-procedural complications such as hemolytic anemia after doxycycline injection in 2 patients, hypoglycemic and metabolic acidosis in 3 neonates, transient hypotension during absolute alcohol instillation and self-limiting skin excoriation secondary to peri-catheter leakage of doxycycline. Other reported complications include permanent vocal cord paralysis after local ethanol injection [374], serious complications after OK-432 injection such as death due to pulmonary embolism [375], deaths due to pulmonary complications after treatment using bleomycin [376, 377] and leukocytopenia due to bleomycin [368].

### Limitations

There are few papers that solely analyzed LM around the cervical airway. Most papers included lesions involving not only the neck but also the craniofacial and other parts of the body and reported LM with different properties such as cystic and mixed types. In addition, definition of cavernous lesions and methods of sclerotherapy (injection techniques, number of injections) were not similar among papers, and differences in these backgrounds must be taken into consideration to evaluate the effectiveness of sclerotherapy.

<Summary>

The CQ, “Should sclerotherapy be performed in infancy for a patient with head and neck LM affecting the airway?”, was evaluated from the viewpoints of responses (prognosis (survival rate or mortality), decrease in size, symptoms, cosmetic improvements) and complications. Since there have been some reports on the risk of respiratory distress due to LM around the airway in infants, and therapeutic intervention is necessary even in infants when the risk is high or they have already developed symptoms. Such intervention is made by sclerotherapy or surgery, and as surgical resection is associated with the risk of more serious complications than sclerotherapy, intervention by less invasive sclerotherapy is recommended. Sclerotherapy is considered to be very effective because of high regression rate of the lesion and symptom/function-improving effect. However, its effect varies depending on the disease type, somewhat less effective in the cavernous and mixed types than in the cystic type. Furthermore, when it was performed for the lesions around the airway, it may be associated with the risk of exacerbation of airway obstruction symptoms due to reactive enlargement of the lesion. Thus, we formulated the recommendation, “In LM around the airway, there is the risk of respiratory disturbances from infancy, but airway obstruction is likely to be exacerbated by sclerotherapy. Particularly, when the risk of airway obstruction is judged to be high or when symptoms have appeared, it is proposed to perform sclerotherapy with sufficient preparations including securing the airway.”


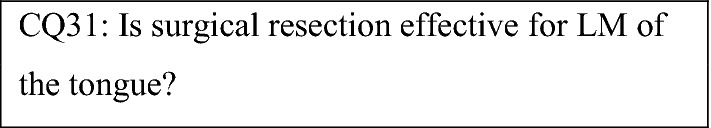


Recommendation:

Surgical resection is effective for reducing the size of the lesion and alleviating symptoms and functional impairment. However, total resection is often difficult, and careful decision is required in consideration of the possibility of complications and recurrence.

Strength of recommendation: 2 (weak)
Evidence: D (very weak)

#### Comments

[Process of preparation of recommendation]

While the tongue is one of the frequent sites of LM, the lesion is often distributed widely over the neck rather than is localized in the tongue. LM of the tongue not only cause cosmetic problems, such as protrusion from the mouth and bleeding, but also readily occupy the oropharyngeal cavity and cause functional problems such as disorder of mouth closing, difficulty in speaking, respiratory disturbances, and impairment of oral food intake. These conditions are treated at departments including plastic surgery, oral surgery, otorhinolaryngology, and pediatric surgery. LM of the tongue are treated by surgical resection or sclerotherapy, but comprehensive evaluation of the condition of individual cases including the distribution of the lesion in the tongue, involvement of other areas and cyst components, and vascular distribution in addition to general information, such as the risk of complications and recurrence in each treatment, is necessary.

Therefore, the CQ, “Is surgical resection effective for LM of the tongue?” was formulated, and the present knowledge about the effectiveness of surgical resection of the lesion, particularly, by partial glossectomy was summarized.

<Literature search and screening>

As a result of search, 29 papers in Japanese and 76 papers in English (75 from PubMed, 1 from Cochrane) were subjected to primary screening. Of these papers, 2 in Japanese and 10 in English were subjected to secondary screening concerning this CQ. They included 1 retrospective cohort study, but most other papers were case series or case reports. Consequently, in the evaluation of this CQ, the results and discussion of the cohort study and each case series were integrated.

<Review of observational studies (case series)>

The effectiveness of surgical resection of LM of the tongue was evaluated from the viewpoints of resectability of the lesion, symptoms, function, and cosmetic improvements as elements of responses as well as complications and recurrence.

### Results of review

1. Responses
2. Resectability of the lesion

Twenty-four cases of tongue lesions treated by surgical resection alone were reported in 4 papers. Catalfamo et al. [378] performed surgical resection of localized masses including normal structures with a margin of 1 cm in the horizontal direction and reported that the size of tongue lesions was reduced in 8 (88.9%) of the 9 patients.

Concerning large lesions impossible to resect totally, Boardman et al. [358] reported 13 cases of partial surgical resection, but several operations were often necessary to reduce lesion size. A total of 2 case have been reported [379, 380], and the lesion size was reduced in both. Although differences were observed in re-enlargement after surgery, they are discussed in detail in “(2) Complications”.

In 1 case report, sclerotherapy was performed 15 times, but the lesion size could not be reduced, and surgical resection was performed, eventually resulting in a favorable outcome without recurrence [381].

According to a report of 89 cases of head and neck LM by Lei et al. [291], the outcome was excellent in 73 (82%) and good in 16 (18%) although it was not a report of cases of tongue lesions alone. They included 43 cases of tongue lesions.

In addition, a few papers [382–385] that suggested the effectiveness of combinations of surgical resection with sclerotherapy and laser therapy were observed. Wiegand et al. [383] classified the disease into 4 stages according to the area of involvement and reported that the stage can be a prognostic factor. Surgery was effective, and complications were rare, when the lesion was localized in the superficial layer and part of the muscle layer. Surgical resection can also be effective, but complete resection is difficult, when the lesion extends over the entire muscle layer or to the tongue base and neck. Therefore, partial resection is often repeated and combined with laser therapy and sclerotherapy, but the recurrence is observed very frequently, and the results did not contradict the reports mentioned below in the section of the recurrence rate [291, 358].

B. Symptoms

A wide variety of symptoms have been reported depending on the site of the mass, and they include tongue discomfort, bleeding, pain, and difficulty in oral feeding [386]. Roy et al. [387] reported that bleeding from the tongue surface, pain, and eating difficulty were alleviated by cauterization.

C. Functions

In most patients who exhibited functional impairment, the lesions were so extended that they were no longer indications for one-time surgical resection. Large masses located at sites such as the tongue base cause respiratory disturbances, swallowing disorders, and difficulty in speech. According to the report by Azizkhan et al. [385], oral intake of normally cooked food became possible in 14, and normal vocalization became possible in 8, of the 21 patients with tongue base lesions. In addition, 5 of the 17 patients who needed tracheotomy were weaned.

D. Cosmetic improvements

Objective evaluation of cosmetic effects is also difficult.

Azizkhan et al. [385] reported that, of the 20 patients, excluding 1 with severe deformity who died, deformity of structures around the tongue, such as the mandible and maxilla, was mild in 6, moderate in 5, and severe in 9. There have been a few reports that cosmetic improvements were also observed in patients who showed a reduction of the tongue size by surgical resection, but objective evaluation is insufficient.

2. Complications

Although the properties of the lesions are unclear in some papers, facial nerve paralysis, vagus nerve paralysis, infection, hematoma, seroma, salivary leakage, ruptured suture, and skin flap necrosis have been reported as complications of the facial region. There have also been reports of temporary complications such as pain and hemorrhage.

3. Recurrence

There have been a few postoperative evaluations reporting that no reactivation that clinically required treatment was observed. Lei et al. [291] reported greater details: Recurrence was observed in 21 (23.6%) of 89 patients and was more frequent in those aged less than 1 year, those with lesions in the oral cavity/face, those with lesions at 3 or more sites, and those with microcystic lesions. According to Boardman et al. [358], LM of the tongue recurred in 12 (48%) of 28 patients, more often than other head and neck lesions. As factors related to this more frequent recurrence of lingual LM, more frequent involvement of other regions, such as the floor of mouth, and a high percentage of microcystic lesions (70%) have been suggested. Of the 2 patients treated by surgical resection alone, 1 who underwent resection of the middle part of the tongue showed no re-enlargement for 1 year or longer after surgery [379], but surgery was repeated 3 times in the 1 who underwent marginal resection [380]. This patient who underwent repeated resections also showed no re-enlargement although the time of the last resection is unclear.

### Limitations

In some papers, surgical resection was combined with other treatments [381–385, 387], lesions in other areas such as the neck were included [291], and the lesion types were unknown. The lack of standardization of subjects and uniformity of the definition or time of recurrence must be considered in the evaluation of the effectiveness of surgical resection.

<Summary>

Many papers suggest that surgical resection is effective for reducing the size of lingual LM. However, in patients with large lesions, lesions extending to structures other than the tongue, and microcystic lesions, several resections or combination of resection with other treatments such as sclerotherapy and laser therapy were necessary, and the recurrence rate tended to be higher. While a few papers referred to symptoms, functional outcome, and cosmetic improvements, none showed a high level of evidence, and the evidence was insufficient for general discussion of the effectiveness of surgical resection.

Therefore, concerning the effectiveness of surgical resection for LM of the tongue, “Surgical resection is effective for reducing the size of the lesion and alleviating symptoms and functional impairment. However, total resection is often difficult depending on the distribution of the lesion, and careful decision is required in consideration of the possibility of complications and recurrence.” was proposed as a draft recommendation.


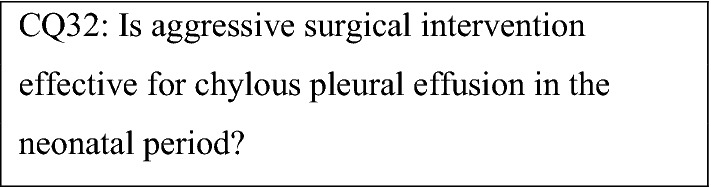


Recommendation:

For chylous pleural effusion refractory to conservative treatments, surgical procedures, such as pleurodesis, ligation of the thoracic duct, and pleuroperitoneal shunting, may be effective.

Strength of recommendation: 2 (weak)
Evidence: D (very weak)

#### Comments

[Process of determining recommendation]

Primary chylous pleural effusion during the neonatal period is often refractory and can be fatal. Thoracic drainage is performed for respiratory insufficiency due to accumulation of pleural effusion, followed by conservative treatments, such as nutritional therapy, steroid, and octreotide therapy, conducted primarily by neonatologist until resolution of chylous pleural effusion.

In refractory cases that do not respond to these conservative therapies, surgical intervention, such as ligation of the thoracic duct and pleurodesis, may be performed. However, no sufficient consensus has been obtained concerning their effects. To evaluate problems, such as at what point surgical intervention should be made and whether aggressive surgical intervention is effective for such a condition, the CQ, “Is aggressive surgical intervention effective for chylous pleural effusion in the neonatal period?”, was formulated, and the knowledge available at present was summarized.

<Literature search and screening>

As a result of search, 98 papers in Japanese and 264 papers in English (262 from PubMed, 2 from Cochrane) were subjected to primary screening. Of these papers, 8 in Japanese and 9 in English were subjected to secondary screening concerning this CQ. They included none with a high level of evidence, such as a systematic review or RCT that evaluated surgical treatment, and all papers were case series or case reports. Consequently, the results and discussion in each of the case series judged to be useful for the preparation of the draft recommendation were integrated although they were weak as the evidence for the evaluation of this CQ.

<Review of observational studies (case series)>

The literature concerning the effectiveness of surgical treatment for chylous pleural effusion in the neonatal period was reviewed from the viewpoints of responses and complications.

### Results of review

1. Responses

Surgical treatment for neonatal chylothorax is performed in patients who respond insufficiently even to thoracic drainage in addition to nutritional therapy using MCT milk or total parenteral nutrition or drug treatment such as octreotide administration.

The methods for surgical intervention found by the present literature review included ligation of the thoracic duct and pleuroperitoneal shunting as well as pleurodesis with OK-432 administration, intrathoracic infusion of fibrin, and povidone-iodine administration, and some patients diagnosed in utero underwent pleuro-amniotic shunting. Cases in which mildly invasive treatments, such as thoracoscopic ligation of the thoracic duct and intrathoracic fibrin application, have been reported in addition to those who underwent thoracic duct ligation by thoracotomy.

Treatments that were performed before surgery and their periods were not uniform. In addition, since there are cases that developed chylous pleural effusion after surgery and those of congenital chylothorax, the diversity of the patient background must be taken into consideration in the efficacy evaluation.

Among the surgically treated cases, those in whom chylous plural effusion disappeared, respiratory symptoms were alleviated, and weaning from the respirator became possible have been reported [388, 389]. In addition, the absence of recurrence or reactivation is considered to be a point. [388–391] There were reports that chylous pleural effusion after thoracic surgery was resolved by drainage alone. Cleveland et al. [392] considered conservative treatments, such as total parenteral nutrition, octreotide, and diuretic administration, to be the best and, observing that, of the poor responders, the mortality was 80% in 5 who continued to be managed by conservative treatments but 0% in 4 who underwent additional surgery, reported that surgical treatment contributed to the reduction of the mortality. According to the guidelines for the treatment of chylous thoracic effusion by Buttiker et al. [393], conservative treatment should be continuing for about 3 weeks but should be abandoned thereafter because of the risk of nutritional disturbance, increased susceptibility to infection, and liver disorders. However, Kaji et al. [394] reported that it is difficult to set a clear period of conservative therapy, because the effectiveness and success rate of surgical treatment are unclear.

2. Complications

As complications due to sclerosing agents, fever and increased inflammatory reaction due to the administration of OK-432 as well as pulmonary abscess and temporary flaccidity and protrusion of the upper abdominal region considered to have been due to intercostal nerve damage have been reported. While chyle leakage in the abdominal cavity was noted in a patient who underwent pleuroperitoneal shunting, there were no reports of fatal complications.

### Limitations

Surgical treatment was performed in most reported cases when responses to conservative therapy were not obtained. Therefore, it must be assumed that the results of evaluation of this CQ are based on data concerning the effectiveness of surgery performed with conservative therapy.

<Summary>

The literature was reviewed concerning the effectiveness of aggressive surgical intervention for neonatal chylous thoracic effusion from the viewpoints of responses and complications, but no objective study with a high level of evidence was found. In most reported cases, surgical treatment was performed when responses to conservative treatments were poor. Therefore, it is difficult to compare surgery with other therapies, and the evaluation of the period of conservative treatment before surgery remains insufficient. However, there was a paper that proposed surgical intervention after attempting conservative treatments for 3 weeks as a standard.

Thus, surgical intervention for neonatal chylous pleural effusion is characterized at present as an approach that may be effective but should be evaluated when the condition is not improved by other treatments, and “Surgical procedures, such as pleurodesis, ligation of the thoracic duct, and pleuroperitoneal shunting, may be effective for chylous pleural effusion refractory to conservative treatments.” is proposed as a draft recommendation.


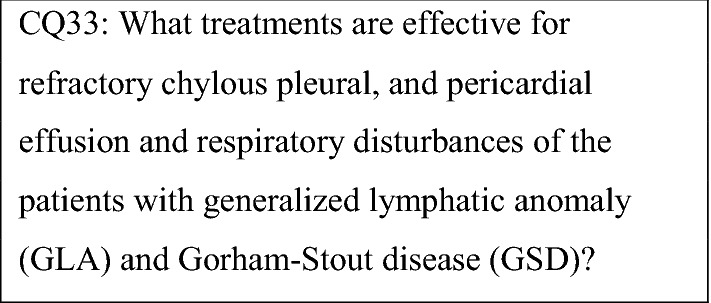


Recommendation:

While treatments including surgery, sclerotherapy, radiotherapy, nutritional therapy, and drug therapy are conducted, there is presently no effective treatment with a high level of evidence. Treatments should be selected in consideration of complications and adverse effects according to individual symptoms.

Strength of recommendation: 2 (weak)
Evidence: D (very weak)

#### Comments

[Process of preparation of recommendation]

GLA and GSD are refractory diseases that cause a wide variety of symptoms in the entire body and are difficult to diagnose and treat. The investigation by the Health and Labour Sciences Research group (Ozeki group) carried out by 2013 showed that the mortality is particularly high when the patients had thoracic lesions.

Among the thoracic symptoms, chylous pleural effusion/pericardial effusion are often refractory and occasionally fatal. While information about the disease is extremely limited because of its rareness, case reports are being globally accumulated as chronic cases are managed on an outpatient basis, and as severe cases are treated intensively.

Presently, no radical treatment for these refractory diseases is known, but the CQ, “What treatments are effective for refractory chylous pleural, and pericardial effusion and respiratory disturbances of the patients with GLA and GSD?” was formulated to compile the knowledge about what treatments are effective as it is a problem of clinical importance.

<Literature search and screening>

As a result of search, 208 papers in Japanese and 617 papers in English (598 from PubMed, 19 from Cochrane) were subjected to primary screening. Of these papers, 2 in Japanese and 25 in English were subjected to secondary screening concerning CQ 37. They included no studies with a high level of evidence, such as a systematic review and RCT, and all were reports of 1-2 cases. Therefore, the evaluation of this CQ was performed by integrating the results and discussion in case series judged to be useful for the preparation of the draft recommendation despite the lack of evidence.

<Review of observational studies (case series)>

The effectiveness of various treatments for refractory GLA and GSD was evaluated according to the prognosis and the presence or absence of improvement in imaging findings, improvement in symptoms, improvement in airway obstruction, enlargement of the lesion, regression, treatment-related complications, recurrence, and reactivation.

### Conditions of patients

The cause of chylous pleural and pericardial effusion is lymphorrhea from lymphatic vessel tissue lesions that have primarily invaded the mediastinum and pleura, and lymphorrhea from osteolytic lesions of the ribs and vertebrae was also observed. Respiratory disturbances were caused by pleural effusion, chylous pleural effusion, pericardial effusion, and direct invasion of the mediastinum and lungs.

### Results of review

As surgical treatments for chylous pleural effusion, procedures, such as thoracentesis, thoracic drainage, ligation of the thoracic duct, and pleural decortication, have been performed, and local lesions were surgically resected. In most cases, thoracentesis and thoracic drainage were performed, but chyle leakage was not resolved. As for complications, there were patients who developed hypovolemic shock and required blood transfusion and catecholamine administration or supplementation of albumin, immunoglobulin, and clotting factors [395–397]. While chylous pleural effusion was controlled in some patients who underwent ligation of the thoracic duct [397–408], the treatment was performed in combination with other surgical procedure or radiotherapy in all cases [400, 402, 408]. There was 1 case that showed improvement in respiratory disturbance [406]. As complications of ligation of the thoracic duct, splenomegaly and lymphorrhea [405] and left-sided pleural effusion [397, 405] have been reported. In the patients who showed marked improvements in chylous pleural effusion [395, 405, 408] after pleural decortication [395, 396, 401, 403–405, 408, 409], the procedure was performed in combination with other surgical treatments or sclerotherapy, and there was no mention about complications. There were cases that showed marked improvements in chylous pleural effusion [396, 400, 405, 408] among those who underwent surgical resection of local lesions including splenectomy [396, 397, 400, 405, 408, 410–412], but the procedure was performed in combination with other surgical treatments in most of them. Hemorrhage was reported as a complication [410]. Among other treatments, pleuroperitoneal shunting [403] and lung transplantation [413] were performed, and alleviation of respiratory disturbance was noted in the patient who underwent lung transplantation.

As a surgical treatment for pericardial effusion, pericardiocentesis was performed [396, 414–416], and pericardial fenestration was performed when pericardial effusion could not be controlled by pericariocentesis [396, 416]. There was no mention about complications.

As sclerotherapy, pleurodesis was performed using OK-432, talc, and minocycline [395, 397–399, 404, 408, 411, 416–418]. There were patients who responded markedly to sclerotherapy alone and sclerotherapy combined with surgical procedures such as pleural decortication or local radiotherapy. There was no mention about complications of sclerotherapy.

There have also been reports [399, 400, 402–404, 410–412, 415, 416, 418–420] on local (e.g., lesion area, thoracic duct region) and thoracic radiotherapy for chylous pleural effusion and local lesions, and marked responses of chylous pleural effusion and responses of respiratory symptoms were noted, but other treatments were performed concomitantly in some patients. Radiation pneumonitis has been reported as a complication [416].

Concerning nutritional therapy, fasting, high-calorie infusion, and MCT diet have been performed alone or in combinations, but few cases that showed alleviation of chylous pleural effusion were observed [395, 396, 398–400, 403, 405, 408, 421].

For drug therapy against chylous pleural effusion, drugs including interferon α, propranolol, anticancer agents (e.g., vincristine), bisphosphonate, octreotide, steroid, sirolimus, and low-molecular-weight heparin were used. Interferon α was used most frequently [395–398, 400, 401, 403, 415, 421], and marked improvement in chylothorax was reported in 5 cases. Of these cases, interferon α was used with propranolol in 1 [395] and with low-molecular-weight heparin and local radiotherapy (15 Gy) in 1 [400]. As for complications of drug therapy using interferon α, there were reports of fever, nausea, and headache [421] and thrombocytopenia and hepatic toxicity [397]. There was no report of improvement in chylous pleural effusion by the use of steroid [395, 399, 403, 415] or octreotide [395, 397, 398, 400, 403, 405] alone. Concerning other drug therapies, only a few cases have been reported with no improvement in chylous pleural effusion. One case that showed regression of mediastinal invasion of GLA and alleviation of respiratory disturbance by sirolimus treatment has been reported [414], and hypertension was noted as a complication. In drug therapy for pericardial effusion, diuretics were used for conservative therapy [400].

### Limitations

Although cases that responded to various therapies have been reported, treatments are often performed in combinations, and the evaluation of the effectiveness of each treatment alone is difficult at this point.

<Summary>

Treatments effective for GLA and GSD presenting with refractory chylous pleural effusion, pericardial effusion, and respiratory disturbances were evaluated by a review of the literature, which was primarily case reports. Various treatments, such as surgery, sclerotherapy, radiotherapy, nutritional therapy, and drug therapy, have been performed, but, there was no study with a sufficient number of cases and a high level of evidence because of the rareness of the disease and diversity of symptoms. Although cases that responded to various treatments have been reported, treatments are frequently performed in combinations, and the evaluation of the effectiveness of individual therapies is difficult at present. Sirolimus (a mTOR inhibitor) is considered promising as a drug for this disease, and some clinical trials are currently under way in Japan and abroad.

In clinical situations, these diseases are not recognized as indications of various drug therapies by the Japanese health insurance system, and the therapeutic effects of other treatments are also uncertain. Therefore, the above treatments cannot be recommended, but we propose that treatments “should be selected in consideration of complications and adverse effects according to individual symptoms”. It is necessary to evaluate the invasiveness, complications, and adverse effects, and select the treatments judged to be appropriate for each case.

## Conclusion

The practice guidelines for vascular anomalies have been prepared as the evidence-based guidelines for the management of vascular anomalies.
